# CSF1R-dependent macrophages in the salivary gland are essential for epithelial regeneration following radiation-induced injury^#^

**DOI:** 10.1126/sciimmunol.add4374

**Published:** 2023-11-03

**Authors:** John G. McKendrick, Gareth-Rhys Jones, Sonia S. Elder, Erin Watson, Wouter T’Jonck, Ella Mercer, Marlene S. Magalhaes, Cecilia Rocchi, Lizi M. Hegarty, Amanda L. Johnson, Christoph Schneider, Burkhard Becher, Clare Pridans, Neil Mabbott, Zhaoyuan Liu, Florent Ginhoux, Marc Bajenoff, Rebecca Gentek, Calum C. Bain, Elaine Emmerson

**Affiliations:** 1 The Centre for Regenerative Medicine, The University of Edinburgh, 5 Little France Drive, Edinburgh, EH16 4UU, UK; 2 The Centre for Inflammation Research, The University of Edinburgh, Queen’s Medical Research Institute, 47 Little France Drive, Edinburgh, EH16 4TJ, UK; 3 The Centre for Reproductive Health, The University of Edinburgh, Queen’s Medical Research Institute, 47 Little France Drive, Edinburgh, EH16 4TJ, UK; 4 Institute of Physiology, University of Zurich, Zurich, Switzerland; 5 Institute of Experimental Immunology, University of Zurich, Zurich, Switzerland; 6 The Roslin Institute & Royal (Dick) School of Veterinary Studies, The University of Edinburgh, Easter Bush Campus, Midlothian, EH25 9RG, UK; 7 Shanghai Institute of Immunology, Department of Immunology and Microbiology, Shanghai Jiao Tong University School of Medicine, Shanghai 200025, China; 8 Singapore Immunology Network, Agency for Science, Technology and Research, Singapore 138648, Singapore; 9 Translational Immunology Institute, SingHealth Duke-NUS Academic Medical Centre, Singapore, Singapore; 10 Centre d’Immunologie de Marseille-Luminy, Aix Marseille Université UM2, INSERM, U1104, CNRS UMR7280, 13288 Marseille, France

## Abstract

The salivary glands often become damaged in individuals receiving radiotherapy for head and neck cancer, resulting in chronic dry mouth. This leads to detrimental effects on their health and quality of life, for which there is no regenerative therapy. Macrophages are the predominant immune cell in the salivary glands and are attractive therapeutic targets due to their unrivalled capacity to drive tissue repair. Yet, the nature and role of macrophages in salivary gland homeostasis, and how they may contribute to tissue repair following injury is not well understood. Here, we show that at least two phenotypically and transcriptionally distinct CX3CR1^+^ macrophage populations are present in the adult salivary gland, which occupy anatomically distinct niches. CD11c^+^CD206^–^CD163^–^ macrophages typically associate with gland epithelium, whereas CD11c^–^CD206^+^CD163^+^ macrophages associate with blood vessels and nerves. Using a suite of complementary fate mapping systems, we show that there are highly dynamic changes in the ontogeny and composition of salivary gland macrophages with age. Using an *in vivo* model of radiation-induced salivary gland injury combined with genetic or antibody-mediated depletion of macrophages, we demonstrate an essential role for macrophages in clearance of cells with DNA damage. Furthermore, we show that epithelial-associated macrophages are indispensable for effective tissue repair and gland function following radiation-induced injury, with their depletion resulting in reduced saliva production. Our data, therefore, provide a strong case for exploring the therapeutic potential of manipulating macrophages to promote tissue repair and thus minimize salivary gland dysfunction after radiotherapy.

## Introduction

Therapeutic radiation remains a mainstay for cancer treatment. Recent advances in the delivery of radiation have minimized off-target side-effects, however, healthy tissues that remain within the therapeutic field often also receive high doses of radiation, leading to cellular damage and organ dysfunction (1). Thus, the salivary glands are often inadvertently damaged following radiotherapy for head and neck cancer, and this results in xerostomia, or chronic dry mouth (2). While considerable efforts have been made to understand the side effects of radiation injury on the salivary glands, there is presently no regenerative therapy for this debilitating condition (1).

Macrophages have long been considered key cells in the tissue repair process and, in recent years, the idea of macrophages as therapeutic targets following radiation injury has become a realistic possibility (3). However, macrophages are incredibly plastic cells that can adopt phenotypic and functional states depending on signals received from their environment, and the nature of these signals is known to change across the course of injury and repair (4, 5). Thus, understanding the composition of tissue macrophage compartments, and if it changes following injury, is key to determining whether macrophages could be targeted therapeutically. Application of single cell technologies, such as single cell RNA sequencing (scRNA-seq), have revealed the diversity of macrophages across and within tissues in both mouse and human (6–8). Diversity can arise from discrete environmental imprinting in distinct anatomical locales, but also from changes in the ontogeny of macrophages. It is now clear that many murine tissue macrophages arise from embryonic progenitors that seed tissues during development (reviewed in *9*). However, the capacity of these embryonic-derived macrophages to persist in adulthood appears to be niche-specific. For example, while brain microglia derive exclusively from embryonic progenitors, macrophages in the choroid plexus are replaced by hematopoietic stem cells (HSC)-derived monocytes (10); and although all gut macrophages are initially derived from embryonic sources, those in the mucosa are replaced by HSC-derived monocytes while muscularis macrophages appear to be longer lived (11–14). Importantly, macrophages of differing origins have been shown to play functionally-distinct roles in the context of infection and fibrosis in the lung (14, 15), in cardiac regeneration after myocardial infarction (16), and in the CNS in response to systemic endotoxin (17).

While the diversity and ontogeny of macrophages across most tissues has been described (6), those in the salivary glands remain relatively poorly defined (18). To address this, we used immunophenotyping, confocal microscopy and scRNA-seq to show that the mouse submandibular salivary gland (SMG) contains at least two populations of spatially distinct CX3CR1^+^ macrophages defined by their expression of CD206, CD163 and CD11c. Using a combination of lineage tracing approaches, we show that while there is a contribution of embryonic precursors to the SMG macrophage compartment during development, these embryo-derived macrophages are displaced in the late embryonic and neonatal stages by HSC-derived macrophages, which require low-rate replenishment by monocytes throughout adult life. We show that all SMG macrophages rely on signalling via the colony stimulating factor 1 receptor (CSF1R) for their development. Radiation-induced injury leads to alterations in the composition of the macrophage pool, and although immediate post-radiation replenishment occurs through in-situ self-renewal, radiation treatment accelerates long-term macrophage replenishment from monocytes. Lastly, using a combination of approaches to deplete SMG subsets or their subsets, we demonstrate an indispensable role for CD11c^+^CD206^–^CD163^–^ epithelia-associated macrophages in the repair and functionality of the SMG after irradiation. Together, these data highlight the integral role of macrophages during effective salivary gland repair.

## Results

### CSF1R-dependent macrophages dominate the SMG immune compartment

**Figure 1:**
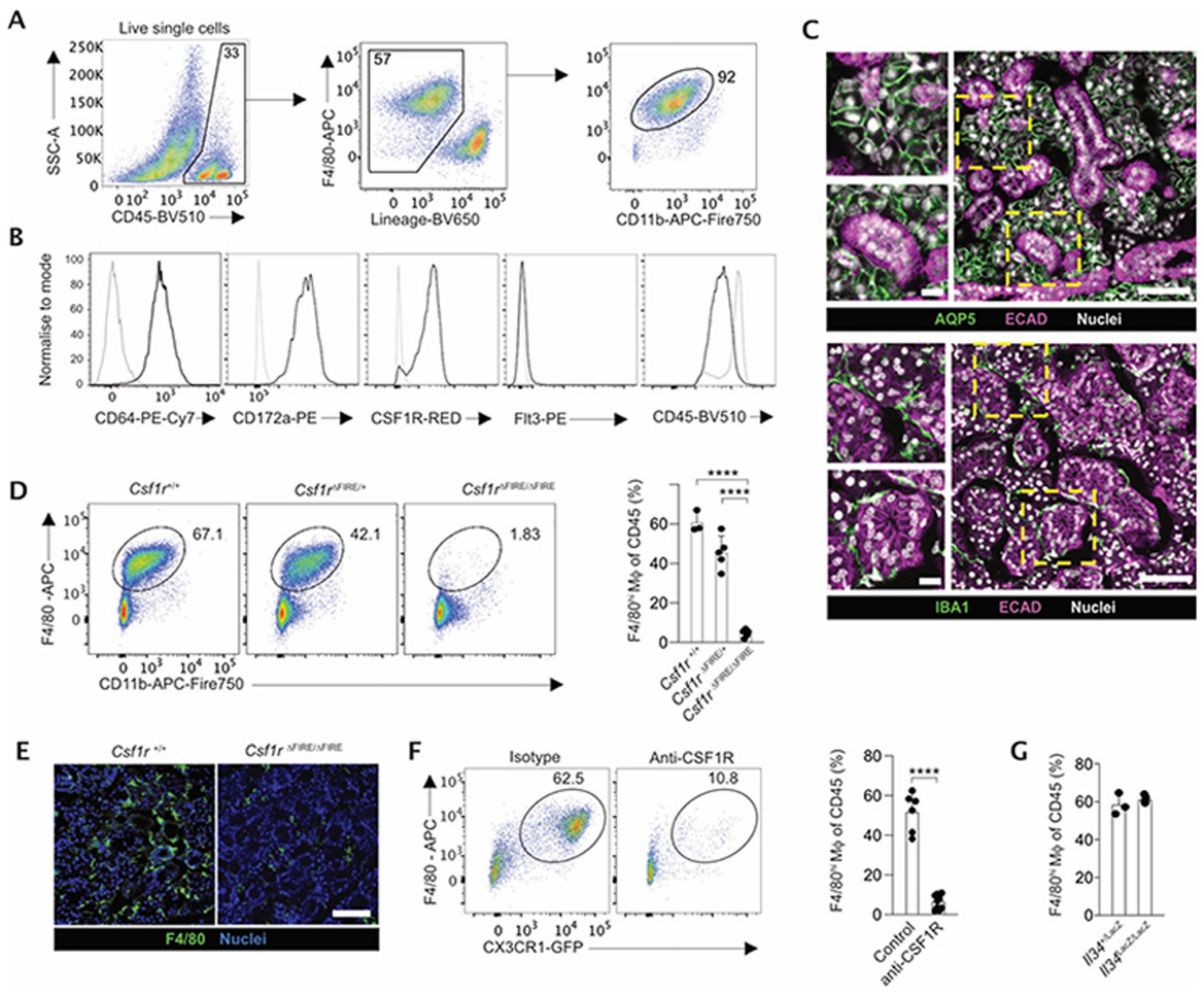
CSF1R-dependent macrophages dominate the SMG immune compartment **A**. Gating strategy for the identification of F4/80^+^ macrophages in the unmanipulated submandibular salivary gland (SMG). **B**. Representative expression of CD64, CD172a, CD45 and Flt3 by F4/80^+^ macrophages obtained from SMG from unmanipulated adult C57BL/6J mice and expression of CSF1R-Red by F4/80^+^ macrophages obtained from SMG from unmanipulated adult *Csf1r*^FRed^ mice. Data are from one of two independent experiments. Grey histograms represent fluorescence minus one (FMO) controls (CD64, CD172a, Flt3), background fluorescence of SMG macrophages from *Csf1r*^WT^ mice (CSF1R-Red) or total leukocytes (CD45). **C**. Representative expression of aquaporin 5 (AQP5) and E-cadherin (ECAD) (upper panels) and ECAD and IBA1 (lower panels) in SMG tissue from unmanipulated adult C57BL/6J mice. Scale bars in large panel = 100μm, magnified insets = 10μm. **D**. Representative expression of F4/80 and CD11b by CD45^+^Lin^–^ live leukocytes from the SMG of unmanipulated adult *Csf1r*^ΔFIRE/ΔFIRE^ mice and their *Csf1r*^+/+^ and *Csf1r*^ΔFIRE/+^ littermates. Graphs show the frequency of macrophages of CD45^+^ leukocytes. Data are from 3 (*Csf1r*^+/+^), 5 (*Csf1r*^ΔFIRE/+^) or 5 (*Csf1r*^ΔFIRE/ΔFIRE^) mice per group and are pooled from 2 independent experiments. ****p<0.0001 (one-way ANOVA with post-hoc Tukey *Q* test) and error bars represent the SD. **E**. Representative expression of F4/80 in SMG tissue of unmanipulated adult *Csf1r*^ΔFIRE/ΔFIRE^ mice and their *Csf1r*^+/+^ littermates. Scale bar = 200μm. **F**. Representative frequency of F4/80^+^CD11b^lo^ macrophages in SMG of adult *Cx3cr1*^+/gfp^ mice administered anti-CSF1R (AFS98) or isotype control for 3 days. Data are from 6 (isotype) or 9 (anti-FCS1R) mice per group and are pooled from two independent experiments. ****p<0.0001 (unpaired Student’s *t* test) and error bars represent the SD. **G**. Frequency of F4/80^+^CD11b^lo^ macrophages in SMG of adult *Il34*^LacZ/LacZ^ mice compared with *Il34*^+/LacZ^ littermate controls. Data are from 3 (*Il34*^+/LacZ^) or 5 (*Il34*^LacZ/LacZ^) mice per group and are from 1 representative experiment of 2.

First, we set out to characterize the macrophage compartment of the naïve murine submandibular gland (SMG), the most well studied of the three major salivary glands, using a combination of flow cytometric analysis and multicolored immunofluorescence. In the adult SMG, we could identify a large population of F4/80-expressing cells that made up the majority of leukocytes (Fig. 1A). Further phenotyping showed that these F4/80^+^ cells expressed CD11b (Fig. 1A), in addition to high and uniform expression of CD172a (SIRPα) (Fig. 1B). These F4/80^+^ cells also expressed high levels of CD64, a marker considered to be expressed by tissue macrophages (19), but lacked expression of Flt3, which is routinely used to define conventional dendritic cells (cDC) (20) (Fig. 1B). SMG macrophages also expressed high levels of CSF1R (Fig. 1B), determined by using a novel CSF1R reporter mouse in which a cassette containing FusionRed fluorescent protein is inserted into the *Csf1r* locus (21). Structurally, the salivary gland is composed of saliva-producing acinar cells, that express both the water channel aquaporin-5 (AQP5) and the epithelial protein E-Cadherin (ECAD), and saliva-transporting ductal cells that express ECAD alone (Fig. 1C; top image). Immunofluorescent analysis showed that macrophages (defined by expression of IBA-1 or F4/80) exist throughout the murine SMG epithelia, and surround both acini, identified by their distinctive circular structure and lower expression of ECAD; and ducts, identified by their closely packed nuclei in a tubular arrangement and confirmed by strong expression of ECAD (Fig. 1C; bottom image and fig. S1A). In keeping with their expression of CSF1R and to determine if SMG macrophages were reliant on CSF1R signalling for their development and/or maintenance, we assessed mice deficient in a super-enhancer region of the CSF1 receptor, termed the *fms*-intronic regulatory element (FIRE) (*Csf1r*^ΔFIREΔ/FIRE^ mice), which selectively impacts CSF1R expression and tissue macrophage development in a variety of tissues (22). Adult *Csf1r*^ΔFIRE/ΔFIRE^ mice had significantly (p<0.0001) fewer F4/80^+^ SMG macrophages compared with *Csf1r*^+/+^ and *Csf1r*^+/ΔFIRE^ littermates (Fig. 1D, E). In contrast, *Csf2ra*^–/–^ mice, which are deficient for the alpha subunit of the receptor for granulocyte-macrophage colony stimulating factor (GM-CSF; also known as CSF2), had normal density of SMG macrophages compared with *Csf2ra*^+/+^ littermates (fig. S1B), ruling out a role for GM-CSF in the development/maintenance of these cells. To complement our findings in *Csf1r*^ΔFIREΔ/FIRE^ mice, we administered an anti-CSF1R blocking antibody (AFS98) to *Cx3cr1*^+/gfp^ mice for three days before assessing macrophage abundance. Treatment caused a marked reduction of F4/80^+^CX3CR1^+^ SMG macrophages, not seen in recipients of the isotype control antibody (Fig. 1F). There are two known ligands for the CSF1R: CSF1 and IL-34. While *Il34*-deficient mice lack Langerhans cells and microglia (23, 24), analysis of adult *Il34*^LacZ/LacZ^ mice showed no difference in the abundance of SMG macrophages when compared with *Il34*^LacZ/+^ littermates (Fig. 1G). Therefore, and in keeping with previously published studies (25, 26), our results demonstrate conclusively that SMG macrophages depend on the CSF1-CSF1R axis for their development.

### Developmentally-related heterogeneity in macrophages exists in the SMG

**Figure 2:**
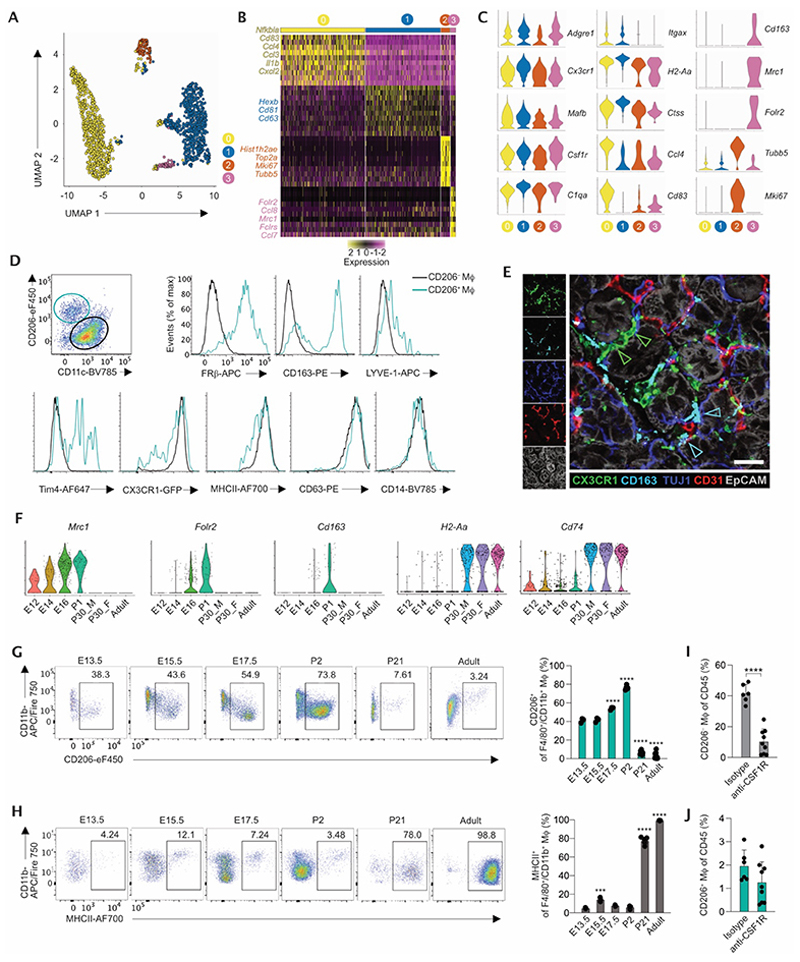
Developmentally-related heterogeneity in macrophages exists in the SMG **A**. Combined UMAP dimensionality reduction analysis of *Adgre1*^+^*Csf1r*^+^ cells from scRNA-seq of SMGs from unmanipulated adult male and female C57BL/6J mice. **B**. Heatmap showing the top 20 most differentially expressed genes by each cluster defined in **A**. and annotated to show genes of particular interest. **C**. Violin plots showing gene expression of curated pan-macrophage and cluster-defining genes from **B**. **D**. Representative expression of CD206, CD11c, FRβ, CD163, LYVE1, Tim4, CX3CR1, MHCII, CD63 and CD14 by F4/80^+^ macrophages obtained from SMG from unmanipulated adult C57BL/6J mice. Data are from one of two independent experiments. **E**. Representative expression of CX3CR1-GFP, CD163, beta-III-tubulin (TUJ1), CD31 and EpCAM in SMG tissue of unmanipulated adult *Cx3cr1*^*gfp/+*^ mice. Scale bar = 25μm. Large image is composed of merged channels, individual channels are presented as smaller images to the left (CX3CR1 = green; CD163 = cyan; TUJ1 = blue; CD31 = red; EpCAM = grey). Green arrowheads point to an example of a CX3CR1^+^ macrophage, cyan arrowheads point to an example of a CD163^+^ macrophage. **F**. Feature plots showing expression of *Mrc1, Folr2, Cd163, H2-Aa* and *Cd74* by *Adgre1*^+^*Csf1r*^+^ cells across a developmental time course from (32). **G-H**. Representative expression of CD206 (**G**) or MHCII (**H**) by F4/80^+^ macrophages from SMG of mice at the indicated ages. Graphs show the mean frequency of CD206^+^ or MHCII^+^ macrophages of total F4/80^+^ macrophages. Data are from 3 (E13.5, 15.5, 17.5), 5 (P1), 7 (P21) and 11 (adult) mice per group and are pooled from 2 independent experiments. *** p<0.001, ****p<0.0001 (one-way ANOVA with post-hoc Tukey *Q* test) compared to E13.5 and error bars represent the SD. **I-J**. Percentage of CD206^–^ (**I**) and CD206^+^ (**J**) macrophages as a proportion of total CD45^+^ cells in mice administered anti-CSF1R (AFS98) or isotype control for 3 days. Data are from 6 (isotype) or 9 (anti-FCS1R) mice per group and are pooled from two independent experiments. ****p<0.0001 (unpaired Student’s *t* test) and error bars represent the SD.

To allow further characterization of these cells in an unbiased manner, we performed single cell RNA-sequencing (scRNA-seq) of non-granulocytic, non-lymphocytic F4/80^+^ myeloid cells (CD3^–^CD19^–^NK1.1^–^Ly6G^–^SiglecF^–^) obtained from unmanipulated male and female adult SMG using the 10X Chromium platform (fig. S2A). Because we also sequenced endothelia and epithelia in this analysis, we identified macrophages, and their putative subsets, on the basis of *Adgre1* (encoding F4/80) and *Csf1r* (fig. S2B) and re-clustered these cells (Fig. 2A). This revealed four clusters of cells with all four clusters found in both male and female SMGs (fig. S2C). All subsets expressed *Cx3cr1* and *C1qa*, as well as the macrophage-specific transcription factor *Mafb* (Fig. 2B, C). Cluster 3 was defined by expression of genes associated with the cell cycle, including *Mki67, Tubb5* and *Top2a*, suggesting these cells represent proliferating macrophages (Fig. 2B, C). While both Cluster 0 and Cluster 1 express *Itgax* (the gene that encodes CD11c; Fig. 2C), Cluster 0 was defined by higher expression of *Cd81, Trem2, Apoe, Cd63 and Hexb*, whereas Cluster 1 expressed higher levels of *Cd14, Cd83, Ccl3, Ccl4, Cxcl2* and *Cxcl10* (Fig. 2B, C). Further analysis showed that there were 378 significant (adj p<0.05) differentially expressed genes (DEGs) when comparing Cluster 0 (219 up) with Cluster 1 (159 up) (Table S1). Gene set enrichment analysis indicated that while Cluster 0 is enriched for genes associated with differentiation, Cluster 1 is enriched for genes associated with antigen presentation (fig. S2D,E). Cluster 3 appeared to be very distinct when compared with the other clusters, and was defined by expression of *Folr2, Mrc1* (encoding CD206; also known as mannose receptor) and *Cd163* (Fig. 2A-C). To validate this heterogeneity, we assessed expression of folate receptor β (FRβ, encoded by *Folr2*), CD206, CD163, CD14, CD63 and MHCII by flow cytometry. We found a small but distinct population of CD206^+^ macrophages that co-expressed FRβ and CD163 (Fig. 2D). Further analysis showed that despite not being detected clearly at mRNA level (fig. S2C), these cells also expressed Tim4 and lymphatic endothelial vessel hyaluronan receptor 1 (LYVE1) at the protein level, albeit at variable or low levels, markers that have been used to define CD206/FRβ-expressing macrophages in other tissues, and termed TLF (Tim4/LYVE-1/FRβ^+^) macrophages (6, 27, 28) (Fig. 2D). Notably, while CCR2 has been shown to define a subpopulation of macrophages across of number of tissues, this was not the case in the SMG at both transcriptional and protein level (fig. S2C, F). Similarly, we did not find *Csf2rb*-defined subsets of macrophages as recently suggested (29) (fig. S2B). We subsequently confirmed the presence of CD163^+^ macrophages in the SMG by immunofluorescence. By spectral imaging, we demonstrated that they are positioned in anatomically discrete niches: CD163-expressing CX3CR1^+^ macrophages were found in close proximity to blood vessels and nerves, as has been previously described in other tissues (30), while CD163^–^CX3CR1^+^ macrophages were found in close contact with the gland epithelium (Fig. 2E). This difference in anatomical location may suggest that these cells play different roles in the gland. CD206^+^ macrophages also lacked expression of CD11c, which was expressed by CD206^–^ macrophages in the adult SMG (Fig. 2D). Despite expression of antigen presentation apparatus (e.g., *H2-Aa*) showing differential expression at mRNA level (Fig. 2C), this did not translate to meaningful differences at the protein level (Fig. 2E), and both subsets were found in close proximity to CD3^+^ T cells (fig. S2G). Similarly, while we detected expression of surface CD14 and intracellular CD63 by flow cytometry, these did not identify discrete subsets of cells amongst CD206^–^ macrophages (Fig. 2D), suggesting that these clusters (Clusters 0 and 1) shared a surface phenotype but exhibit clear transcriptional differences.

To determine if the heterogeneity seen in the adult SMG, and in particular the presence of *Cd163*^+^*Mrc1*^+^*Folr2*^+^ macrophages (6, 27, 28), was apparent throughout SMG development, we examined macrophage heterogeneity in embryonic and neonatal SMG. Gland ontogenesis is initiated at embryonic day 11.5 (E11.5), epithelial branching begins at E13, and luminization and terminal differentiation occurs at E16, forming an organ capable of secretion by postnatal day 7 (P7) (reviewed in *31*). We examined the profile of SMG macrophages across the developmental time course using a publicly available scRNA-seq ‘atlas’ dataset of whole SMG (32). Using a similar approach to the above, we extracted macrophages from this dataset on the basis of *Adgre1* and *Csf1r* expression (fig. S2H, I). While there were relatively low numbers of macrophages in this dataset compared with our scRNA-seq dataset, we could detect differences between macrophages from E14, P1 and adult SMG. Macrophages from E14 and P1 clustered more closely together than adult SMG, suggesting they are more alike (fig. S2H, I). Strikingly, expression of *Mrc1* and *Folr2* appeared to increase during the embryonic stages until P1, yet expression was low or almost absent by 30 days of age and in mature adults (Fig. 2F). Conversely, *H2-Aa* and *Cd74* (the invariant chain of the MHCII complex), which appeared to define Clusters 0 and 1 in our scRNA-seq dataset, were expressed at negligible levels during development, but expressed at high levels by macrophages in the adult SMG (Fig. 2F). Confocal microscopy confirmed the presence of CD163^+^ and CD206^+^ macrophages during embryonic development (fig. S2J, K). Again, we used flow cytometry to validate these scRNA-seq data, showing that CD206^+^ macrophages were present in the embryonic SMG, and came to dominate the neonatal SMG, while only forming a small minority of SMG macrophages by adulthood (Fig. 2G). Of note, we found that CD206/CD163^+^ macrophages were less abundant when enumerated by flow cytometry compared with microscopy, suggesting they may be more difficult to isolate with standard enzymatic protocols. However, the relative loss of CD206/CD163^+^ macrophages with age was also evidenced by microscopy (Fig. 2E and fig. S2J, K). In parallel, expression of MHCII appeared to be induced in the postnatal period, with ~75% of macrophages expressing MHCII by 3 weeks of age (P21), while MHCII is essentially present in all macrophages by adulthood (Fig. 2H), consistent with postnatal acquisition of MHCII by macrophages in other tissues (6). Analysis of adult *Csf1r*^ΔFIRE/ΔFIRE^ mice showed that both CD206-defined macrophage subsets rely on the CSF1R for their development (fig. S2L, although their acute dependence on CSF1 may vary given CD206^–^ macrophages were more markedly affected by short-term anti-CSF1R treatment than their CD206^+^ counterparts (Fig. 2I, J). Thus, dynamic changes to the composition of the CSF1R-dependent SMG macrophage compartment occur in the late embryonic, neonatal and juvenile periods.

### Postnatal switch in the ontogeny of SMG macrophages

**Figure 3:**
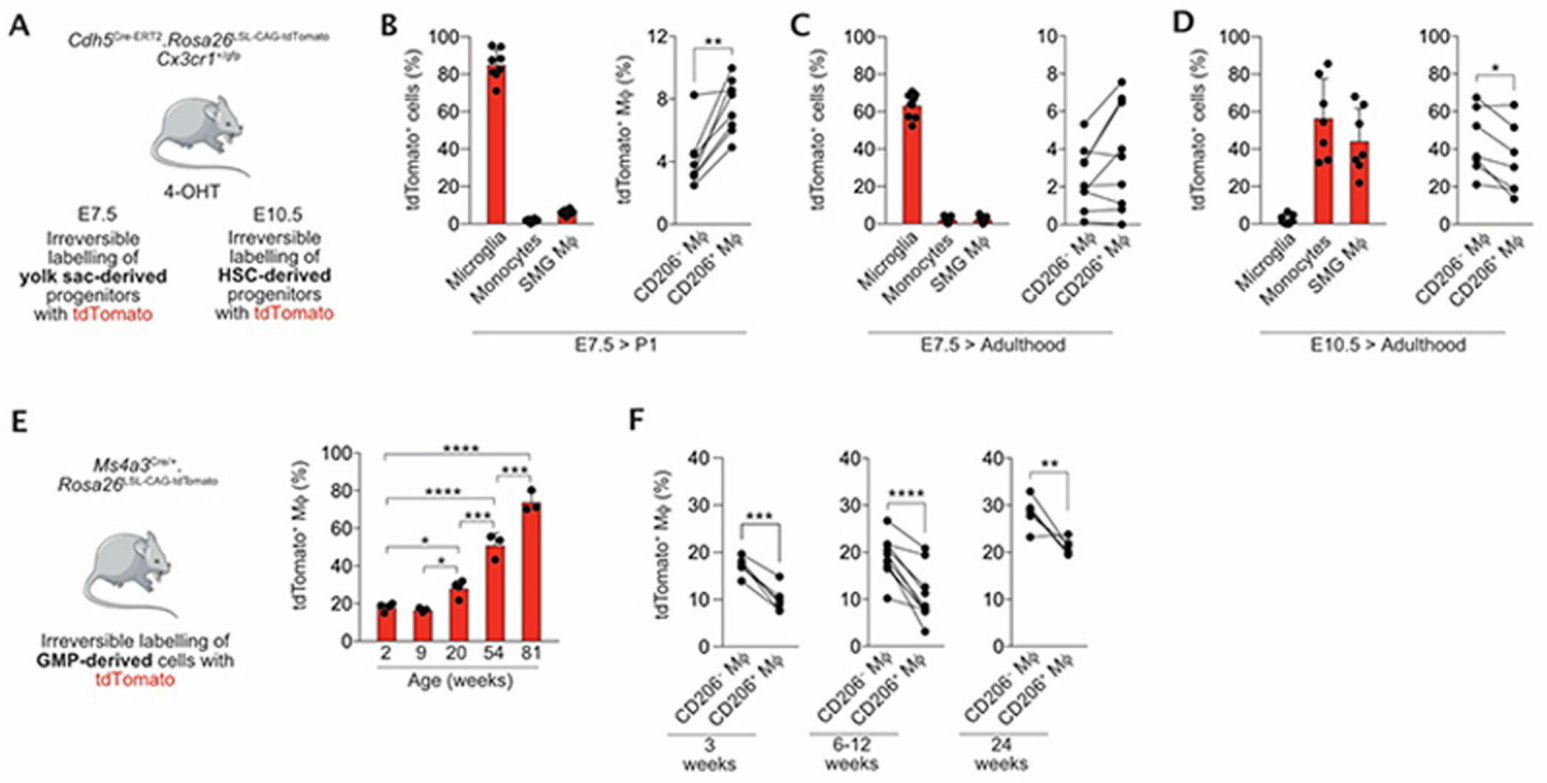
Postnatal switch in the ontogeny of SMG macrophages **A**. Experimental scheme for fate mapping with *Cdh5*^Cre-ERT2^.*Rosa26*^LSL-CAG-tdTomato^.*Cx3cr1*^+/gfp^ mice. **B-C**. The frequency of tdTomato^+^ cells amongst brain microglia, blood monocytes and SMG F4/80^+^CD11b^lo^ macrophages as a total population (*left*) or as CD206-defined subsets (*right*) in the neonatal (P1) (**B**) or adult (**C**) offspring of mice pulsed with 4-hydroxytamoxifen (4-OHT) at embryonic day 7.5 (E7.5). Data are from 8 mice from 3 independent litters (**B**) or 9 adult mice pooled from 2 experiments (**C**). **p<0.01 (One-way ANOVA with Tukey *Q* post-hoc testing). **D**. The frequency of tdTomato^+^ cells amongst brain microglia, blood monocytes and SMG F4/80^+^CD11b^lo^ macrophages as a total population (*left*) or as CD206-defined subsets (*right*) in adult offspring of mice pulsed with 4-OHT at embryonic day 10.5 (E10.5). Data are from 7 mice pooled from 2 experiments. *p<0.05 (One-way ANOVA with Tukey *Q* post-hoc testing). **E-F**. Description of *Ms4a3*^Cre/+^.*Rosa26*^LSL-CAG-tdTomato^ mice (**E**). Graph shows the frequency of tdTomato^+^ cells amongst total F4/80^+^CD11b^lo^ macrophages (**E**) or CD206-defined subsets (**F**) obtained from *Ms4a3*^Cre/+^.*Rosa26*^LSL-CAG-tdTomato^ mice at the indicated ages. Data are from n=4 mice for 2- and 20 week time points, and n=3 for all other time points in E; data are from n=6 mice for 3- and 24 week time points, and n=10 for 6-12 week time points in F. *p<0.05, **p<0.01, ***p<0.001, ****p<0.0001 (One-way ANOVA with Tukey *Q* post-hoc testing). In all graphs symbols represent individual mice and error bars represent the SD.

Given the presence of macrophages in the embryonic SMG and the dynamic changes seen during the neonatal and juvenile periods, we next assessed the ontogeny of SMG macrophages using multiple complementary fate mapping approaches that together delineate their progenitor sources and homeostatic turnover from the adult bone marrow. We first used *Cdh5*^CreERT2/+^.*Rosa26*^CAG-LSL-tdT/+^.*Cx3cr1*^GFP/+^ mice to fate map cells during embryonic development (Fig. 3A). Yolk sac-derived progenitors and definitive haematopoietic stem cells (HSCs) are produced at different sites and developmental stages (33), but due to their endothelial origin, both can be labelled in the *Cdh5*^Cre-ERT2^ model (34) via administration of 4-hydroxytamoxifen at either E7.5 (yolk sac) or E10.5 (HSC) (Fig. 3A). As expected, brain microglia were highly labelled in offspring of mothers pulsed at E7.5 (Fig. 3B, C). In contrast, blood monocytes from the same mice showed only very low levels of labelling. Importantly, we found that, similar to blood monocytes, the total SMG macrophage compartment showed minimal contribution of cells labelled at E7.5 when analyzed in newborn mice (P1) or in 8-12 week old adult mice (Fig. 3B, C). However, analysis of CD206-defined subsets revealed differences in labelling efficiency. Consistent with the presence of CD206^+^ in the embryonic gland, labelling was markedly higher in this fraction compared with CD206^–^ macrophages at P1 (Fig. 3B). By adulthood there was no longer a difference in the presence of yolk-sac derived macrophages in the gland, suggesting that these cells are largely displaced by HSC-derived cells (Fig. 3C). Analysis of the offspring of mothers pulsed at E10.5 supported this finding. In these mice, microglia were very poorly labelled, but circulating monocytes were efficiently labelled, consistent with their derivation from HSCs (Fig. 3D). Macrophages in the SMG of adult (8-12 week old) mice were found to have equivalent labelling to that seen in monocytes, suggesting these too are derived from HSC-derived monocytes by adulthood. We did find a small but significant difference in labelling between CD206-defined macrophage subsets, with lower labelling in CD206^+^ macrophages (Fig. 3D), suggesting either that the rate of replenishment may differ between these subsets or that non-HSC-derived macrophages may persist longer amongst CD206^+^ macrophages.

To test this directly, we next assessed the contribution of monocytes to SMG macrophages at different life stages by examining *Ms4a3*^Cre/+^.*Rosa26*^CAG-LSL-tdTomato^ mice in which granulocyte-monocyte progenitors (GMPs) and their progeny are labelled irreversibly with tdTomato fluorescent protein (Fig. 3E and Fig. S3A). Consistent with their derivation from BM GMPs, circulating monocytes are labelled with high efficiency (>95%) in adult *Ms4a3*^Cre/+^.*Rosa26*^LSL-CAG-tdTomato/+^ mice (35). In contrast, brain microglia show negligible labelling in the same mice (35). Longitudinal analysis of these mice showed that there was slow, but progressive accumulation of tdTomato labelling in the SMG compartment (Fig. 3E), consistent with the accumulation of monocyte-derived cells. In keeping with the differences seen between CD206-defined subsets in the *Cdh5*-based fate mapping, we found the CD206^+^ macrophages were labelled to a lower extent in *Ms4a3*^Cre/+^.*Rosa26*^LSL-CAG-tdTomato/+^ mice than their CD206^–^ counterparts in at all ages examined (Fig. 3F and fig. S3B). However, the rate of replenishment appeared identical between CD206-defined subsets (fig. S3B), suggesting that differences in labelling in *Ms4a3*^Cre/+^.*Rosa26*^LSL-CAG-tdTomato/+^ mice is due to the elevated persistence of non-HSC-derived cells amongst CD206^+^ macrophages.

Taken together, these data demonstrate that embryo-derived macrophages initially seed the SMG but are almost completely replaced by HSC-derived cells, most likely in the neonatal period. These cells become long-lived macrophages, although they are replenished at a low and equivalent rate by BM-derived monocytes.

### Irradiation injury alters the composition and longevity of SMG macrophages

**Figure 4:**
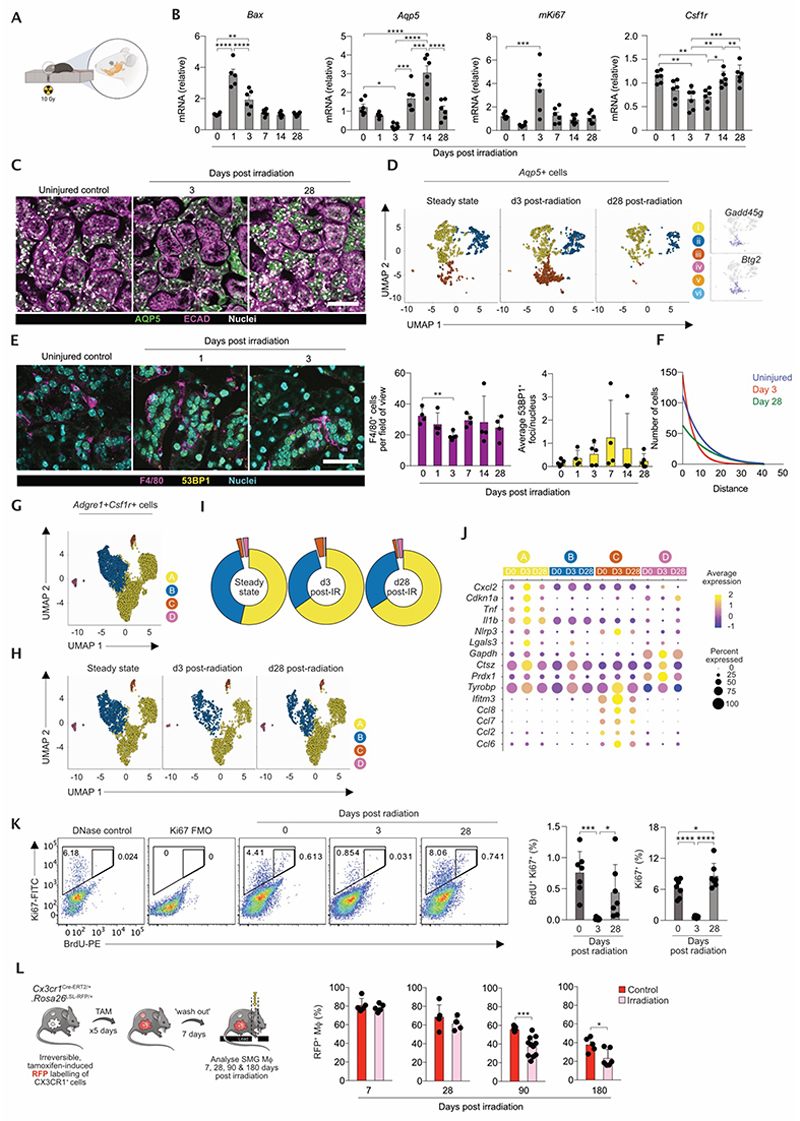
Irradiation injury alters the composition and longevity of SMG macrophages **A**. Schematic of targeted radiation. Image created in BioRender.com. **B**. qPCR analysis of *Bax, Aqp5, mKi67* and *Csf1r* mRNA in total SMG tissue at the indicated time points following radiation induced injury. Data are normalised to mRNA levels in unmanipulated naïve (d0) SMG tissue. Data are from 5-6 mice pooled from 2 independent experiments. *p<0.05, **p<0.01, ***p<0.001, ****p<0.0001 (One-way ANOVA followed by Tukey *Q* post-hoc testing). **C**. Representative expression of aquaporin 5 (AQP5) and E-cadherin (ECAD) obtained from SMG of uninjured mice or mice irradiated 3 or 28 days earlier. Scale bar = 100μm. **D**. UMAP dimensionality reduction analysis of scRNA-seq data from *Aqp5* ^+^ acinar cells split by timepoint. Insets show expression of *Gadd45g* and *Btg2*. **E**. Representative expression of F4/80 and 53BP1 in the SMG of uninjured mice or mice irradiated 1 or 3 days earlier. Scale bar = 25μm. Graphs show the enumeration of F4/80^+^ and 53BP1^+^ cells at the indicated time points following radiation. Data obtained from three fields of view from non-sequential sections from 4-5 mice per timepoint. **p<0.01 (One-way ANOVA with Tukey *Q* post-hoc testing). **F**. Graph showing the distance between macrophages and nuclei containing 53BP1 foci, in the SMG of uninjured mice or mice irradiated 3 or 28 days earlier, measured using nearest neighbour analysis. Data obtained from three fields of view from non-sequential sections from 3 mice per timepoint. Individual graphs are shown in fig. S4E. **G-H**. UMAP dimensionality reduction analysis of scRNA-seq data from 3,478 *Adgre1*^+^ and *Csf1r*^+^ macrophages aggregated across all timepoints (**G**) or split by timepoint (**H**). **I**. Proportion by total number of each macrophage sub-clusters defined in **G** from SMG of unmanipulated mice or mice irradiated 3 or 28 days earlier presented as an exploded pie chart per timepoint. **J**. Bubble plot showing expression of selected genes by macrophage clusters from unmanipulated SMG (D0) or SMG at day 3 (D3) or day 28 (D28) following radiation treatment. **K**. Representative expression of BrdU and Ki67 in F4/80^+^CD11b^lo^ macrophages from SMG at 3, 7 or 28 days post radiation or from unmanipulated mice. Graphs show frequency of BrdU^+^Ki67^+^ F4/80^+^CD11b^lo^ macrophages and Ki67^+^ F4/80^+^CD11b^lo^ macrophages in the SMG at 3 and 28 days following radiation exposure compared with untreated mice (D0). Data are from 7-8 mice per time point pooled from two independent experiments. *p<0.05, ***p<0.001, ****p<0.0001 (One-way ANOVA followed by Tukey *Q* post-hoc testing). **L**. Experimental scheme for fate mapping CX3CR1^+^ cells in adult *Cx3cr1*^Cre-ERT2/+^.*Rosa26*^LSL-RFP^ mice. Graphs show the frequency of RFP^+^ cells amongst F4/80^+^CD11b^lo^ SMG macrophages obtained from *Cx3cr1*^Cre-ERT2/+^.*Rosa26*^LSL-RFP^ mice administered tamoxifen by oral gavage for 5 days and analyzed at the indicated time points following targeted radiation. Data are 5-11 mice pooled from 3 independent experiments. *p<0.05, ***p<0.001 (unpaired Student’s t test). In all graphs symbols represent individual mice and error bars represent the SD.

Having established the heterogeneity and replenishment kinetics of SMG macrophages during homeostasis, we next used our well-validated mouse model of irradiation injury, where the neck and SMGs are irradiated with a single dose of 10Gy while the rest of the body is shielded (Fig. 4A), to characterize how these cells respond following irradiation. This regime is a well-established model of irradiation injury (36–40) and recapitulates the damage experienced by radiotherapy patients (41). As previously reported in the murine sublingual gland (42), transcriptional analysis of whole SMG tissue confirmed early cellular apoptosis and epithelial damage, with a significant increase in pro-apoptotic *Bax* expression at days 1 and 3 post-irradiation, which had returned to baseline levels by day 7 (Fig. 4B). In addition, expression of *Aqp5*, which encodes the water channel protein AQP5, was essentially absent at day 3, before recovering by day 7 and day 28, with a peak of expression at day 14. Consistently, AQP5 protein, localized to the apical membranes of acinar cells, was evident in uninjured SMG (Fig. 4C), whereas expression was aberrant at 3 days post-injury, and epithelial architecture (marked by the epithelial marker ECAD) was clearly disrupted before returning to baseline levels by day 28 (Fig. 4C). Similarly, expression of the acinar cell marker MIST1 (43) followed the same pattern (fig. S4A); the luminal marker claudin-10 (CLDN10) (44) was expressed in an aberrant manner at day 3 (fig. S4B); and vasculature, marked by CD31, was less abundant at day 3 (fig. S4C, as previously reported (45). We then utilized the epithelial data in our scRNA-seq dataset and analyzed transcriptional changes in *Aqp5*^+^ acinar cells. We observed the appearance of a cluster (iii) that was found almost exclusively at day 3 and expressed *Gadd45g* and *Btg2* (Fig. 4D), genes that are known to be involved in DNA damage and the stress response to injury (46, 47). Consistent with exposure to radiation, overall proliferation, as measured by *mKi67* expression at tissue level, was reduced at day 1 but elevated at day 3 before returning to baseline thereafter (Fig. 4B). In parallel, we found significantly (p<0.01) lower levels of *Csf1r* mRNA in SMGs following irradiation, which was restored to previous levels by day 14 (Fig. 4B), suggesting radiation may alter the abundance and/or the composition of the macrophage pool. Consistent with this, enumeration of macrophages by confocal microscopy showed that radiation treatment led to a reduction in the absolute numbers of SMG macrophages, which recovered by day 7 (Fig. 4E). Following radiation injury, F4/80^+^ macrophages appeared to preferentially localize to cells displaying signs of DNA damage, marked by expression of 53BP1 (48) (Fig. 4E). Furthermore, using Nearest Neighbour Analysis (fig. S4D) we confirmed that macrophages more closely associate with ductal cells (those that display DNA damage; 53BP1^+^) at day 3 post-irradiation, when compared with uninjured controls and day 28 post-irradiation (Fig. 4F and fig. S4E). Interestingly, flow cytometric analysis of SMG showed that radiation injury caused little or no changes in expression of canonical markers, including F4/80, CD45 and MHCII (fig. S5A, B). In parallel, there was no measurable recruitment of neutrophils or monocytes, suggesting that radiation treatment did not elicit a marked inflammatory response (fig. S5C). In support of this, expression of mRNA for the pro-inflammatory cytokines IL-1β, IL-6 and TNF did not significantly change across the irradiation time course (fig. S5D). To assess if the transcriptional fingerprint of macrophages changed in the context of irradiation, we collected SMG at days 0, 3 and 28 post-irradiation, and undertook scRNA-seq analysis (Fig. 4G-J). We chose these timepoints to span peak injury (day 3) and injury resolution (day 28). As before, we enriched for specific populations by sorting macrophages, epithelial cells and endothelial cells. Macrophages were again identified on the basis of *Adgre1* (F4/80) and *Csf1r* expression (fig. S5E), and re-clustered (Fig. 4G). Again, this revealed 4 clusters (termed A, B, C, D). Clusters A and B were the two largest clusters and aligned with Cluster 0 and 1, respectively, in our earlier analysis (Fig. 4G). Cluster C represented the CD163^+^CD206^+^ population referred to as Cluster 3 in the previous analysis, while Cluster D denotes a population of cycling macrophages, defined by expression of *mKi67, Stmn1* and *Top2a* (Fig. 4G) and aligned with Cluster 2 in the previous analysis. The most striking effect of radiation treatment was the almost complete loss of Cluster D at day 3 post-radiation, consistent with the known effects of radiation interrupting cell proliferation. Notably, by day 28 the relative abundance of Cluster D had recovered to levels seen at steady state (Fig. 4H, I) and we confirmed this pattern using flow cytometry by measuring BrdU incorporation (2hr pulse-chase) and Ki67 expression (Fig. 4K). Pairwise gene expression analysis revealed that certain changes in gene expression were present across clusters, for example, upregulation of *Tyrobp* and *Ctsz*, genes involved in macrophage adhesion and recruitment in other tissues (49, 50), at day 3 in all four groups (Fig. 4J). However, cluster-specific differences were apparent (Fig. 4J, fig. S5F and Table S2). For instance, Cluster A, which expresses higher levels of *Cxcl2* during physiological conditions, showed upregulation of these transcripts immediately following radiation treatment (day 3), before returning to a baseline expression level at day 28 (Fig. 4J), a result that was validated by transcriptional analysis of FACS-purified CD206/CD163-defined SMG macrophages (fig. S5H). Another prominent feature of this cluster following radiation was expression of *Cdkn1a* (Fig. 4J), which encodes cyclin-dependent kinase 1A (also known as p21), a negative regulator of cell cycle. Expression of CDKN1A is known to confer radio-resistance to epidermal Langerhans cells (51) and Kupffer cells (52), and this could explain the relative preservation of this subset. Following radiation Cluster B displayed elevated expression of *Lgals3* (Galectin-3) and *Tyrobp* (DAP12) (Fig. 4J), genes that are involved in macrophage activation and recruitment (49, 53, 54). Post-radiation Cluster C showed heightened expression of the monocyte chemoattractants *Ccl8, Ccl7, Ccl2 and Ccl6* and the interferon transmembrane protein *Ifitm3* (Fig. 4J), a result confirmed by transcriptional analysis of sorted CD163^+^ cells (fig. S5H). In line with previously published data, this population likely acts to recruit other immune cells, while the release of CC chemokines may also confer radioresistance and preservation of this subset (55). Furthermore, *Ifitm3* is known to play a role in preventing cytomegalovirus (CMV) pathogenesis in the salivary gland by controlling cytokine production (56). Both Cluster A and Cluster C showed elevated expression of *Nlrp3* after injury (Fig. 4J and fig. S5H). Since *Nlrp3* is part of the inflammasome and detects products of damaged cells including ATP and uric acid (57), this result suggests that these cells may play a particular role in the recognition and elimination of damaged cells.

The changes in the macrophage compartment seen through scRNA-seq analysis and, in particular, the almost complete loss of proliferating macrophages at the acute time point analyzed (day 3) prompted us to evaluate whether radiation altered macrophage replenishment kinetics. To this end, we performed pulse-chase fate mapping using *Cx3cr1*^Cre-ERT2/+^.*Rosa26*^LSL-RFP/+^ mice by administering tamoxifen for five days, followed by a ‘wash out’ period of one week to ensure circulating classical monocytes were no longer labelled (Fig. 4L). Mice were then subjected to targeted irradiation or left untreated, and loss of RFP signal amongst macrophages measured as a rate of macrophage replenishment from RFP^–^ monocytes (fig. S5I, J). Interestingly, while radiation treatment led to an initial reduction in the absolute number of macrophages (Fig. 4E), which recovered within the first week, such treatment had no effect on the frequency of RFP-expressing macrophages at this stage. This suggests that the replenishment of macrophages at this point appears to occur independently of blood monocytes (Fig. 4L). When examined at day 28, the repair phase, although loss of RFP^+^ macrophages could be detected when compared with day 7, this loss was similar between control and irradiation (Fig. 4L). However, by 3 months there was significantly (p<0.001) greater loss of RFP-labelled SMG macrophages in irradiated mice compared with controls, a phenotype that remained evident at 6 months post-irradiation (Fig. 4L). However, despite this elevated loss of RFP following irradiation, labelling did not differ between CD206-defined subsets (fig. S5L). Extending the ‘wash out’ period to 3 weeks to provide additional confidence that residual RFP labelling in blood monocytes was not responsible for maintenance of RFP^+^ macrophage persistence in the first 4-6 weeks post-irradiation, gave results consistent with the 1 week wash out period (fig. S5I-K). Taken together these data suggest that there are subset-specific responses to radiation treatment, that initial recovery in macrophage numbers occurs through *in situ* proliferation and that elevated macrophage replenishment from blood monocytes occurs in the longer term.

### SMG macrophages are essential for epithelial regeneration following irradiation injury

**Figure 5:**
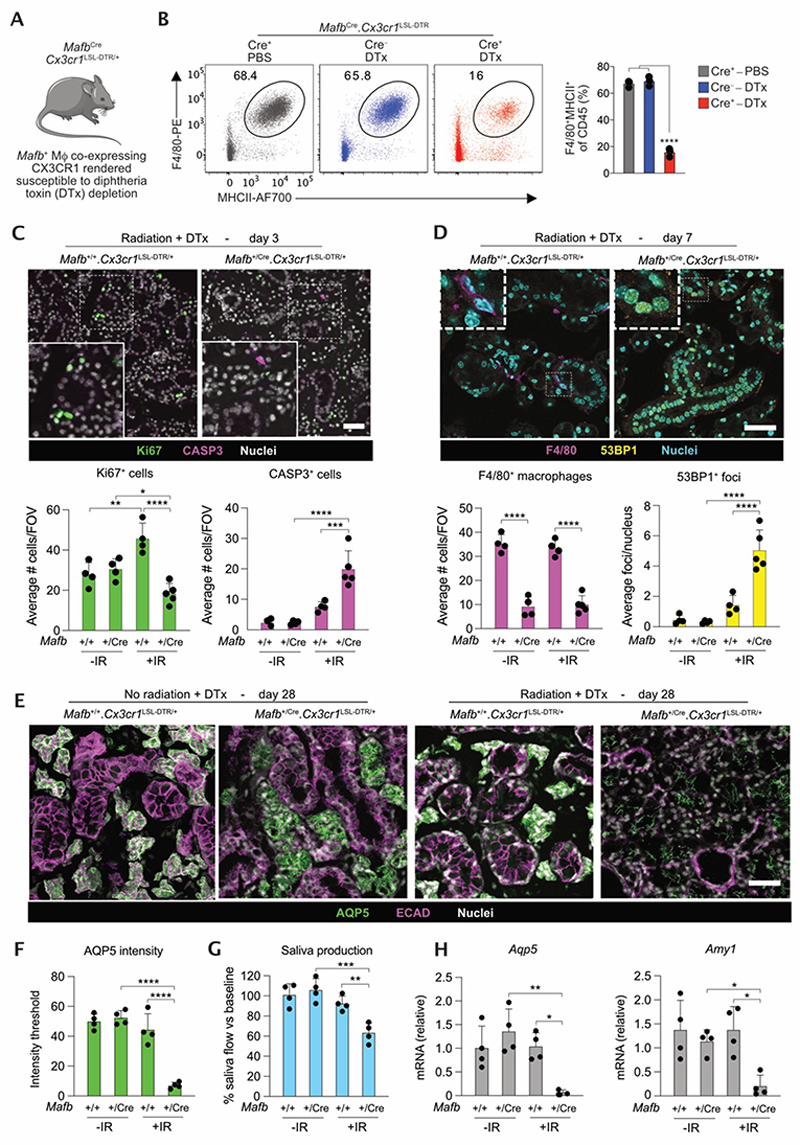
Global *Mafb*-mediated depletion of macrophages hinders SMG regeneration following irradiation injury **A**. Schematic of the *Mafb*^Cre/+^.*Cx3cr1*^LSL-DTR/+^ mouse model. **B**. Representative expression of F4/80 and MHCII by CD45^+^SiglecF^–^Ly6G^–^ leukocytes obtained from *Mafb*^Cre/+^.*Cx3cr1*^LSL-DTR/+^ mice or *Mafb*^+/+^.*Cx3cr1*^LSL-DTR/+^ littermates administered diphtheria toxin (DTx) or saline (PBS). Data are from 3 mice from one of two independent experiments performed. ****p<0.0001 (One-way ANOVA followed by Tukey *Q* post-hoc testing). Symbols represent individual mice and error bars represent the SD. **C**. Representative immunofluorescent images of SMG stained for Ki67 and CASP3 in *Mafb*^Cre/+^.*Cx3cr1*^LSL-DTR/+^ mice or *Mafb*^+/+^.*Cx3cr1*^LSL-DTR/+^ littermates administered diphtheria toxin (DTx) or saline for 5 days before being exposed to 10Gy irradiation, and analyzed 3 days after irradiation. Dashed white line indicates area that has been magnified in inset images (bold white line). Scale bar = 25μm. Graphs show the enumeration of Ki67^+^ and CASP3^+^ cells. Data obtained from three fields of view from non-sequential sections from 4-5 mice per group. *p<0.05, **p<0.01, ***p<0.001, ****p<0.0001 (One-way ANOVA with Tukey *Q* post-hoc testing). **D**. Representative immunofluorescent images of SMG stained for F4/80 and 53BP1 in *Mafb*^Cre/+^.*Cx3cr1*^LSL-DTR/+^ mice or *Mafb*^+/+^.*Cx3cr1*^LSL-DTR/+^ littermates administered diphtheria toxin (DTx) or saline for 5 days before being exposed to 10Gy irradiation, and administered diphtheria toxin (DTx) or saline for a further 3 days before being analyzed 7 days after irradiation. Dashed white line indicates area that has been magnified in inset images (bold white line). Scale bar = 25μm. Graphs show the enumeration of F4/80^+^ cells and 53BP1^+^ foci. Data obtained from three fields of view from non-sequential sections from 4-5 mice per group. ****p<0.0001 (One-way ANOVA with Tukey *Q* post-hoc testing). **E**. Representative immunofluorescent images of SMG stained for AQP5 and ECAD in *Mafb*^Cre/+^.*Cx3cr1*^LSL-DTR/+^ mice or *Mafb*^+/+^.*Cx3cr1*^LSL-DTR/+^ littermates exposed to 0Gy or 10Gy irradiation and administered diphtheria toxin (DTx) or saline from day 17 onwards, every 2 days and analyzed 28 days after irradiation. Scale bar = 25μm. **F**. Enumeration of AQP5 intensity in *Mafb*^Cre/+^.*Cx3cr1*^LSL-DTR/+^ mice or *Mafb*^+/+^.*Cx3cr1*^LSL-DTR/+^ littermates exposed to 0Gy or 10Gy irradiation and administered diphtheria toxin (DTx) or saline from day 17 onwards, every 2 days and analyzed 28 days after irradiation. Data obtained from three fields of view from non-sequential sections from 4 mice per group and error bars represent the SD. ****p<0.0001 (One-way ANOVA with Tukey *Q* post-hoc testing). **G**. Quantification of saliva production in *Mafb*^Cre/+^.*Cx3cr1*^LSL-DTR/+^ mice or *Mafb*^+/+^.*Cx3cr1*^LSL-DTR/+^ littermates exposed to 0Gy or 10Gy irradiation and administered diphtheria toxin (DTx) or saline from day 17 onwards, measured at 27 days after irradiation and presented as a percentage of the baseline measurement. Data are from 4 mice per group and error bars represent the SD. **p<0.01, ***p<0.001 (One-way ANOVA followed by Tukey *Q* post-hoc testing). **H**. qPCR analysis of *Aqp5* and *Amy1* mRNA in total SMG tissue in *Mafb*^Cre/+^.*Cx3cr1*^LSL-DTR/+^ mice or *Mafb*^+/+^.*Cx3cr1*^LSL-DTR/+^ littermates exposed to 0Gy or 10Gy irradiation and administered diphtheria toxin (DTx) or saline from day 17 onwards, every 2 days and analyzed 28 days after irradiation. Data are normalised to mRNA levels in SMG tissue from *Mafb*^+/+^.*Cx3cr1*^LSL-DTR/+^ littermates exposed to 0Gy. Data are from 4 mice per group and error bars represent the SD. *p<0.05, **p<0.01 (One-way ANOVA followed by Tukey *Q* post-hoc testing).

Finally, to determine if SMG macrophages contribute to tissue repair after radiation injury, we used two complementary *in vivo* depletion systems. We first used *Mafb*^Cre/+^:*Cx3cr1*^LSL-DTR/+^ mice, in which all *Cx3cr1*-expressing macrophages are rendered susceptible to diphtheria toxin (DTx), in order to temporally and selectively deplete macrophages in the SMG (Fig. 5A). As shown earlier, all subsets of SMG macrophages express both *Mafb* and *Cx3cr1* (Fig. 2C). We first demonstrated that we could achieve efficient depletion of SMG macrophages using this model. Following two doses of DTx, we observed a significant (p<0.0001) reduction in F4/80^+^ macrophages in the otherwise healthy SMG (Fig. 5B). Using this methodology, we then combined macrophage depletion with radiation injury. Immunofluorescence analysis revealed fewer epithelial Ki67^+^ cells in the absence of macrophages at 3 days post-radiation (Fig. 5C), while CASP3^+^ apoptotic epithelial cells were elevated (Fig. 5C). In parallel, where macrophage depletion had been efficient, as defined by areas devoid of F4/80^+^ cells, DNA damage marked by 53BP1^+^ foci in the cell nuclei was elevated at day 7 post-radiation (Fig. 5D), consistent with the role of macrophages in clearing damaged and dying cells. Studies in mice have shown that after irradiation injury the salivary gland tissue goes through an initial regenerative phase, which ultimately fails in the long term (58–60), mirroring what occurs in humans following radiotherapy (41, 61, 62). We therefore extended our studies to examine the roles of macrophages in the initial regenerative phase by determining the effects of macrophage depletion up to the point at which many indices of damage and inflammation have subsided, and repair is under way (day 28) (39, 60, 63, 64). To this end, we administered DTx every other day from day 17 to day 27, undertook saliva measurements at day 27 and analyzed the SMG at day 28. We confirmed that IBA1^+^ and CD163^+^ macrophages were both depleted in this model via immunofluorescent analysis, while non-myeloid CD45^+^ cells (CD45^+^IBA1^–^) remained unaltered (fig. S6A, B), as did circulating monocyte subsets (fig. S6C, D). Immunofluorescent analysis revealed that macrophage depletion hindered the restoration of salivary gland structure following irradiation injury. Specifically, while the structural and functional markers AQP5 (acinar cells) and ECAD (all epithelia, but high in ductal cells) have returned to a level comparable with uninjured SMG by 28 days in the presence of macrophages (Fig. 5E), macrophage depletion led to aberrant patterning of AQP5 and irregular ductal structures 28 days after injury (Fig. 5E, F and fig. S6E. Given that AQP5 is necessary for water transfer and is the only aquaporin to play a major role in the salivary secretion process (65), the lack of AQP5 here demonstrates that macrophage depletion severely impacts on secretory function. This was confirmed with the finding that stimulated saliva production was significantly (p<0.001) reduced following IR when coupled with macrophage depletion (Fig. 5G). Indeed, ablation alone without irradiation injury also results in structural alterations (Fig. 5E), albeit to a lesser extent, indicating that SMG macrophages may also support homeostatic maintenance of SMG structural cells. While macrophage depletion coupled with irradiation did not have an effect on overall gland weight (fig. S6F), expression of genes which represent function, including aquaporin-5 (*Aqp5*), Amylase 1 (*Amy1*) and Mucin-10 (*Prol1*) and the epithelial adhesion marker E-cadherin (*Cdh1*) were markedly reduced at day 28, while the acinar specification marker *Sox10* was unchanged (Fig. 5H and fig. S6G.

**Figure 6:**
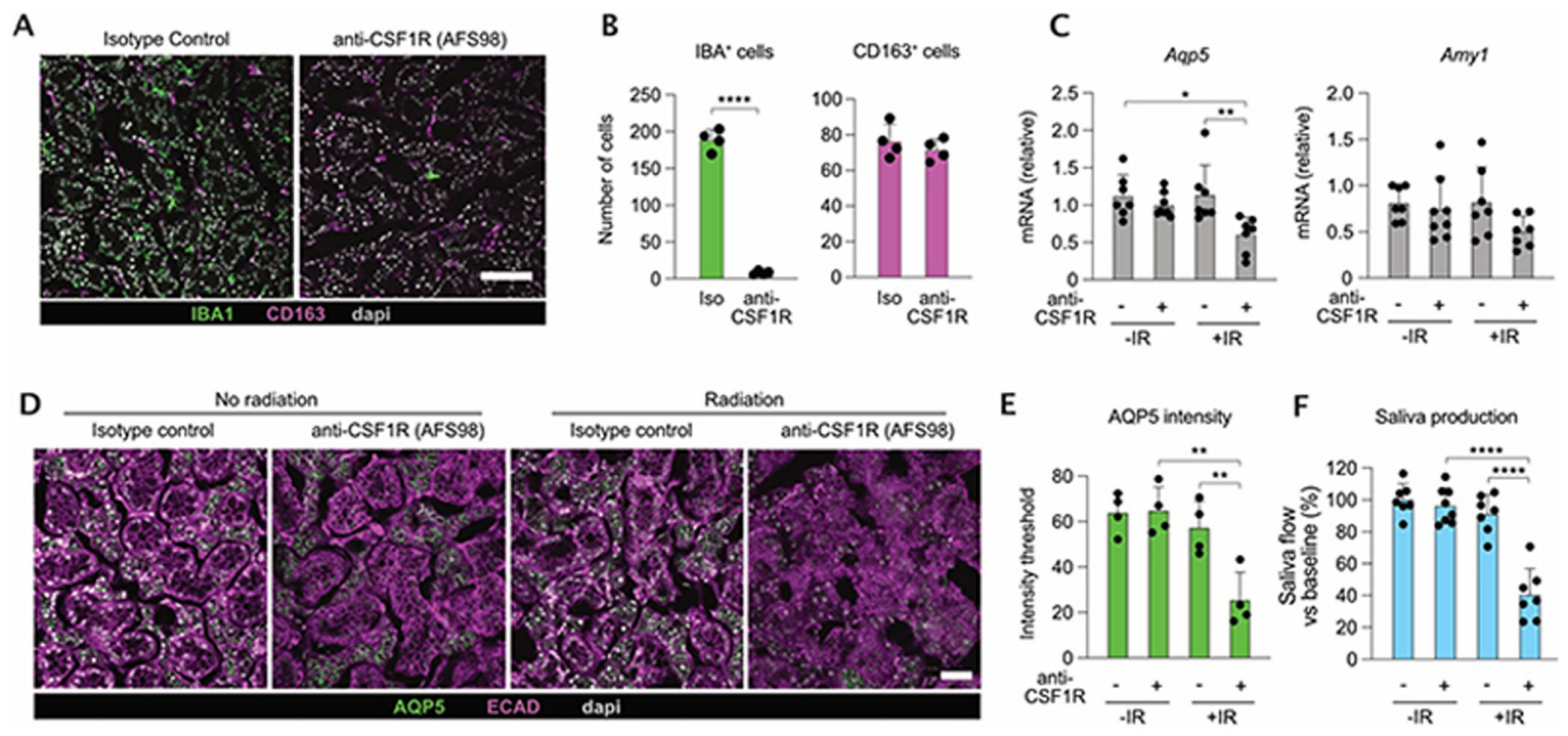
Selective depletion of epithelial-associated macrophages prevents effective repair of SMG following irradiation injury **A-B**. Representative immunofluorescent images of SMG stained for IBA1 and CD163 in C57BL/6 mice exposed to 0Gy or 10Gy irradiation before being administered anti-CSF1R (AFS98) or isotype control and analyzed 28 days after irradiation. Scale bar = 200μm (**A**). Graphs in (**B**) show the enumeration of IBA1^+^ and CD163^+^ cells. Data obtained from three fields of view from non-sequential sections from 4 mice per group and error bars represent the SD. ****p<0.0001 (One-way ANOVA with Tukey *Q* post-hoc testing). **C**. qPCR analysis of *Aqp5* and *Amy1* mRNA in total SMG tissue in C57BL/6 mice exposed to 0Gy or 10Gy irradiation before being administered anti-CSF1R (AFS98) or isotype control and analyzed 28 days after irradiation. Data are normalised to mRNA levels in SMG tissue from mice administered isotype control and exposed to 0Gy. Data are from 7-8 mice per group and error bars represent the SD. *p<0.05, **p<0.01 (One-way ANOVA followed by Tukey *Q* post-hoc test). **D**. Representative immunofluorescent images of SMG stained for AQP5 and ECAD in C57BL/6 mice exposed to 0Gy or 10Gy irradiation before being administered anti-CSF1R (AFS98) or isotype control and analyzed 28 days after irradiation. Scale bar = 25μm. **E**. Enumeration of AQP5 intensity in C57BL/6 mice exposed to 0Gy or 10Gy irradiation before being administered anti-CSF1R (AFS98) or isotype control and analyzed 28 days after irradiation. Data obtained from three fields of view from non-sequential sections from 4 mice per group and error bars represent the SD. **p<0.01 (One-way ANOVA with Tukey *Q* post-hoc testing). **F**. Quantification of saliva production in C57BL/6 mice exposed to 0Gy or 10Gy irradiation before being administered anti-CSF1R (AFS98) or isotype control, measured at 27 days after irradiation and presented as a percentage of the baseline measurement. Data are from 7-8 mice per group and error bars represent the SD. ****p<0.0001 (One-way ANOVA followed by Tukey *Q* post-hoc testing).

To extend the observations in the *Mafb*^Cre/+^:*Cx3cr1*^LSL-DTR/+^ system and in lieu of genetic systems which selectively target CD206-defined macrophage subsets, we exploited the differential susceptibility to anti-CSF1R treatment to target macrophage subsets, as has been used previously (66, 67). CD206/CD163^+^ macrophages were not affected by short-term anti-CSF1R treatment, whereas CD206/CD163^–^ macrophages were significantly (p<0.0001) reduced compared with recipients of isotype control antibody (Fig. 6A, B). Importantly, anti-CSF1R treatment coupled with irradiation led to reduced expression of *Aqp5* and *Cdh1* in whole SMG tissue (Fig. 6C and fig. S7C), which was paralleled by altered AQP5 and ECAD patterning and overall failure to restore salivary gland structure after irradiation injury (Fig. 6D, E and fig. S7A). Furthermore, gland weight and stimulated saliva production was significantly (p<0.001 and p<0.0001 respectively) reduced 28 days after irradiation injury in mice treated with anti-CSF1R compared with isotype controls (fig. S7B and Fig. 6F).

Taken together, these data demonstrate that macrophages are crucial at both early and later stages of SMG repair, with epithelial-associated CD11c^+^CD206^–^CD163^–^ macrophages responsible for supporting epithelial regeneration after irradiation injury.

## Discussion

Macrophage heterogeneity and ontogeny in many tissues has been studied extensively in recent years, yet the salivary glands have remained largely unexplored despite containing a dense network of macrophages (68). Here we have used a combination of approaches to demonstrate that there is considerable heterogeneity within the salivary gland macrophage compartment throughout the life course, resulting in part from changes in the ontogeny of these cells but also from niche-specific imprinting. Furthermore, we show that salivary gland macrophages are crucial for effective tissue repair following radiation-induced injury.

Previous work has leveraged scRNA-seq technologies to chart the cellular composition of the salivary gland (29, 32, 69). However, most of these studies have sought to create a cellular ‘atlas’ of the salivary gland and thus have lacked sufficient resolution to comprehensively and specifically profile the macrophage compartment. Our targeted scRNA-seq provided sufficient resolution to identify four clusters of macrophages which fell into three phenotypically discrete subsets identifiable by flow cytometry: CD163^+^CD206^+^, CD163^–^CD206^–^ and proliferating macrophages (Ki67^+^). During revision of our manuscript, another study describing SMG macrophage heterogeneity was published (29). While both studies describe macrophage heterogeneity, the approaches used to define this heterogeneity are quite different. Zhao et al. (29) define heterogeneity on the basis of *Csf2rb*, which encodes the β subunit of the receptor for GM-CSF, and suggest dependence of this *Csf2rb*^**+**^ subset on innate lymphoid cell (ILC)-derived GM-CSF (29). In contrast, we found no heterogeneity on the basis of expression or reliance on GM-CSF receptors. In our study, CD206^+^CD163^+^ macrophages expressed Tim4, LYVE-1, albeit at low and variable levels, and FRβ, aligning them with so-called “TLF” (Tim4 and/or LYVE-1 and/or FRβ) expressing macrophages found across many different tissues by the Epelman group (6, 27, 28). Interestingly, this is in disagreement with Zhao et al. (29) where the authors conclude that *Folr2* (FRβ), *Lyve1* and *Timd4* are not expressed in any macrophage subset. Clusters 0 and 1 appeared to represent discrete transcriptional states of the CD11c^+^CD206^–^CD163^–^ subset. This characterization is consistent with work by the Stein group where CD11c-YFP reporter mice were used to visualize SMG macrophages (68). The hypothesis that SMG macrophages can be partitioned on the basis of CD11c expression is also consistent with a recent study (70), although we propose that positive identification of CD11c^–^ macrophages on the basis of CD206/CD163 (or FRβ) expression is a superior way to characterise the SMG compartment. Our transcriptional profiling also supports the notion that CD11c^+^CD206^–^CD163^–^ macrophages may interact with T cells in the SMG, given their high and constitutive expression of *Cxcl10*, and expression of CXCR3 by tissue resident memory CD8^+^ T cells adjacent to macrophages in the SMG (68). Future studies ascertaining the role of T cell-macrophage interactions will be fundamental to better understand the kinetics of salivary gland regeneration and degeneration.

Longitudinal analysis across the life course demonstrated that CD206^+^CD163^+^ macrophages dominate the developing and neonatal SMG but form only a minor fraction of the adult SMG macrophage compartment, a finding consistent with the study by Lu *et al*. (70). Previous work has failed to reach a consensus on the replenishment kinetics of SMG macrophages. For instance, while global *Ccr2* deficiency has been used to support the idea that SMG macrophages require no replenishment from classical CCR2-dependent monocytes (71), others have used CCR2 antagonism to reach opposite conclusions (70). By using state-of-the-art genetic lineage tracing, we show that although embryonic progenitors contribute to the initial seeding of the SMG macrophage compartment, these are displaced by HSC-derived macrophages, and low-level contribution of monocytes is needed to maintain the SMG macrophage compartment during adulthood, a finding reproduced using *Ms4a3*^*Cre*^ reporter mice from three institutions. In the spectrum of macrophage replenishment (72), SMG macrophages appear to have similar kinetics to red pulp splenic macrophages (35). Notably, the rate of displacement of embryonic-derived macrophages appeared to be lower amongst CD206^+^ macrophages than the CD206^–^ subset. This could reflect a difference in growth factor dependence, although our analysis of *Csf1r*^ΔFIRE/ΔFIRE^ mice suggests that both CD206-defined subsets depend on CSF1R for their development. Interestingly, CSF1R blockade in adult mice had a more marked effect on CD206^–^/CD163^–^ macrophages compared with their CD206/CD163-expressing counterparts, suggesting differential acute reliance on CSF1R. Notably, we also ruled out a role for GM-CSF and the CSF1R ligand IL-34 in SMG macrophage homeostasis. Taken together with previous work showing a deficit of salivary gland macrophages in *Csf1r* knockout rats (26), similar to that in the *Csf1*^op/op^ mouse (25), which has a naturally occurring inactivating mutation in the *Csf1* gene, our data demonstrate a key role for the CSF1-CSF1R axis in controlling SMG macrophage homeostasis. This is particularly pertinent given that: CSF1 promotes branching morphogenesis of the mouse SMG (73); CSF1 is expressed in response to Hedgehog (Hh) signalling in the SMG (74); and that acinar cells are reported to express CSF1 (75). Together these indicate epithelial-macrophage communication. Moreover, this macrophage-epithelial communication is likely to be bidirectional as depletion of SMG macrophages in *Mafb*^Cre/+^.*Cx3cr1*^LSL-DTR/+^ mice in the absence of radiation treatment led to alterations in the epithelial compartment. Understanding the nature of this crosstalk is a key aim of future work.

In this study we show that radiation-induced injury leads to a subtle shift in SMG macrophage subsets and a striking loss of a proliferative subset. Surprisingly, macrophage repopulation following injury occurred independently of monocytes, suggesting proliferative capacity must be regained relatively quickly following initial injury. Whether all macrophages possess identical capacity to proliferate, or if proliferative subsets may exhibit “stemlike” characteristics (76) is still not known and will require novel transgenic systems. Since we observed a loss of these proliferating macrophages in the early stages after radiation injury, and given the demonstration that proliferative resident macrophages are essential for islet cell proliferation in the pancreas (77), for example, understanding more about these subsets will be integral for future studies. In the long-term macrophage replenishment is accelerated by radiation treatment, suggesting that radiation may cause long-term changes to the macrophage niche, making it less able to support macrophage longevity. Our model of radiation-induced injury allowed us to assess macrophage function in the immediate response (day 3) and during tissue repair (day 28). Crucially, we demonstrate that global depletion of CX3CR1^+^ SMG macrophages in the initial days after irradiation injury leads to an accumulation of DNA damage, failure of epithelial recovery and a loss of tissue functionality. Complementary experiments showed that macrophages play a key role in supporting tissue repair at day 28 after injury. In particular, our data support a role for macrophages in orchestrating epithelial repair and restoration of the secretory function of acinar cells. In contrast, Zhao, *et al*. (74) conclude that depletion of macrophages by clodronate does not affect functional markers or impair saliva production after irradiation injury (74). However, given the very small population of macrophages analyzed in their study (less than 5% of total macrophages), and the complicated nature of action of clodronate liposomes (which leads to a partial and transient depletion only) this is perhaps not surprising (78). In contrast, we show, by two complementary approaches, that macrophage depletion has a significant effect on salivary gland regeneration after injury. While our data suggests that it is the epithelial-associated macrophages that are essential for epithelial regeneration, we cannot rule out the importance of the CD206/CD163^+^ nerve-associated macrophages, particularly given the well-described necessity of nerve signalling in salivary gland regeneration (42). Furthermore, we cannot rule out a role for GM-CSF in controlling macrophage differentiation during and following injury. Indeed, understanding the factors that drive macrophage specialisation, how these change following radiation, and determining how the niche is altered following injury should better inform how macrophages can be targeted therapeutically in the context of irradiation damage.

## Materials and Methods

### Study Design

We performed phenotypic and transcriptomic analysis of submandibular macrophages across development, homeostasis and following irradiation injury, using immunofluorescence and flow cytometric analysis and scRNA-sequencing. Fate mapping techniques were used to explore macrophage ontogeny and replacement kinetics. An *in vivo* irradiation model was used to ascertain the role of radiation injury on macrophage kinetics. All mice were randomised into experimental groups and all imaging and associated analysis were blinded. Experimental replicate details are provided in the figure legends.

### Mouse studies

All procedures adhered to ARRIVE guidelines and were approved by the UK Home Office and performed under PPLs PB5FC9BD2, PP0860257, PP1871024. Mice of both sexes were used. Sex and age of mice is noted for each experiment in the relevant figure legend. Transgenic mice used in this study are listed in Table S3.

### Lineage tracing studies

Labelling of CX3CR1^+^ cells was achieved by administering *Cx3cr1*^Cre-ERT/+^.*Rosa26*^LSL-RFP/+^ mice with 5mg tamoxifen (Sigma Aldrich) in 100μl sunflower oil (Sigma Aldrich) by oral gavage on 5 consecutive days, followed by 7 days or 21 days of no treatment (‘washout’) before further manipulation (e.g. irradiation). Blood sampling was performed to assess RFP recombination in blood monocytes during the washout period. For *Cdh5*^*Cre-ERT2*^ fate mapping, WT female mice aged 6-10 weeks were subjected to timed matings with *Cdh5*^*Cre-ERT2+/-*^ or *Cdh5*^*Cre-ERT2+/+*^ *Rosa*^*tdT/tdT*^ *Cx3cr1*^*gfp/gfp*^ males. Successful mating was judged by the presence of vaginal plugs the morning after, which was considered 0.5 days post-conception. For induction of reporter recombination in the offspring, a single dose of 4-hydroxytamoxifen (4OHT; 1.2mg) was delivered by intraperitoneal (i.p) injection to pregnant females at E7.5 or E10.5. To counteract the adverse effect of 4OHT on pregnancy, 4OHT was supplemented with progesterone (0.6mg). In cases when females could not give birth naturally, pups were delivered by C-section and cross-fostered with CD1 females.

### Anti-CSF1R experiments

Anti-CSF1R antibody (AFS98) was generated as previously described (67, 85). Male C57BL/6 mice were administered with intravenous (i.v) injection of 2mg/mL anti-CSF1R antibody over three consecutive days for homeostatic experiments (250 µg per mouse per day) or every other day over 11 days for post-injury experiments (400 µg per mouse on day 17, 200 µg per mouse on days 19, 21, 23, 25, 27). Mice were assessed the day after the final administration.

### Genetic depletion of *Cx3cr1*^+^ cells

Conditional depletion of *Cx3cr1*^+^ *cells* was achieved by injecting *Mafb*^*Cre/+*^.*Cx3cr1*^*LSL-DTR/+*^ mice with 200ng Diphtheria toxin (DTx; Sigma Aldrich, D0564) in 100 μl sterile saline intraperitoneally (IP) 3x per week. For homeostatic assessment of depletion, mice were examined 24hrs after the final DTx dose. When combined with radiation treatment, mice received DTx every other day for 5 days before irradiation (single dose of 10Gy) and were examined at days 3 and 7 post-irradiation, or mice underwent irradiation before receiving DTx every other day for 10 days from day 17 post-irradiation, and were examined at day 28 post-irradiation.

### Radiation-induced SG injury

Mice were anaesthetized using 1mg/kg Medetomidine Hydrochloride (Dormitor) and 75mg/kg ketamine (Ketavet) in 0.9% saline (Thermo Fisher Scientific). Mice were irradiated using a single ^137^Cs source in a Shepherd Mark-I-68A irradiator (JL Shepherd & Associates), with only the neck exposed and the rest of the head and body shielded with lead, as previously described (42). Non-irradiated control mice were not anaesthetized. After a 20 minute period of anaesthesia, mice were given 1mg/kg of reversal agent Antisedan and were allowed to recover on a heat pad before returning to normal housing. Subsequently, mice were provided with soft diet and DietGel® (Clear H_2_O) *ad libitum*. Mice were euthanised at 1, 3, 7, 14 or 28 days or 3 or 6 months post-irradiation.

### BrdU administration

For cell cycle analysis mice were injected with 1 mg BrdU (Sigma Aldrich) by IP injection 2 hours prior to euthanasia.

### Developmental studies

Embryonic SMGs were collected from embryos at E12.5, E13.5, E14.5, E15.5, E17.5 and postnatal animals at P2, P7 and P21, following timed matings between male and female C57BL/6 mice. Successful mating was confirmed by the presence of a vaginal plug and the morning of discovery deemed E0.5. SMG explants were dissected as previously described (86) and used for immunofluorescent analysis or flow cytometry.

### Cell dissociation Embryonic SMG

SMGs were pooled from multiple mice for E12.5, E13.5, E14.5 (n=3 per sample) given their small size, in order to obtain sufficient material. E15.5, E17.5, P2, P7 and P21 glands were analyzed individually. SMGs were digested in 500μl RPMI-1640 containing 5% fetal calf serum (FCS; Sigma Aldrich), 6µl Collagenase-II (23mg/mL) (Sigma Aldrich), 6µl hyaluronidase (40mg/mL) (Sigma Aldrich) and 37µl 0.1M CaCl_2_ for 10 minutes in a shaking incubator at 100 rpm at 37°C. Tissue was subsequently centrifuged at 400G for 5 minutes at 4°C. The supernatant was discarded and the pellet resuspended in 500μl of HBSS (Lonza) containing 1% BSA (Sigma Aldrich) and 2mM EDTA (Sigma Aldrich) and then passed through a 20µm filter capped 5mL FACs tube using a 5mL syringe with a 25G needle.

### Adult SMG

Each SMG pair (approx. 160mg) was mechanically minced in 500μl of RPMI-1640 containing 2% fetal calf serum (FCS; Sigma Aldrich). 500μl of RPMI + 2% FCS containing 50µl Collagenase-II (100mg/mL) (Sigma Aldrich), 10µl DNAse I (0.1mg/mL) (Roche), 50 µl Dispase II (0.8mg/mL) (Roche), 10ul 10mM HEPES (Sigma Aldrich) and 50µl 0.1M CaCl_2_ was added to each tube and tissue was incubated in a shaking incubator at 100 rpm at 37°C for 30 minutes followed by trituration. The solution was filtered through a 70µm nylon mesh (ThermoFisher), flushed with 2 mL RPMI + 2% FCS and centrifuged at 400G for 5 minutes at 4°C. The cell pellet was resuspended in 1mL of 1x red blood cell lysis buffer (Abcam) for 5 minutes on ice before centrifugation at 400G for 5 minutes at 4°C. The cell pellet was resuspended in 1mL of pre-warmed (37°C) trypsin +0.25% EDTA and incubated at 37°C for 5 minutes before trituration. This step was repeated 3 times until single cells were evident. The solution was centrifuged at 400G for 5 minutes at 4°C and the cell pellet was resuspended in 1mL of Hank’s Balanced Salt Solution (HBSS; Lonza) containing 1% BSA (Sigma Aldrich) and 2mM EDTA (Sigma Aldrich) and then passed through a 20µm filter capped 5mL FACS tube using a 5mL syringe with a 25G needle.

### Isolation of blood monocytes

For analysis of blood monocytes 25µl blood was collected from the mouse tail vein and stored in EDTA-coated tubes on ice until analysis. Blood was transferred to FACS tubes and 500 µl of 1x red blood cell lysis buffer (Biolegend) for 5 minutes on ice before centrifugation at 300G for 3 minutes at 4°C. This step was repeated once more. The cell pellet was resuspended in 50µl PBS containing 1% BSA and 2mM EDTA.

### Isolation of brain microglia

For analysis of microglia, single cell suspensions were obtained from brain tissues via a combination of enzymatic digest and mechanical dissociation. Brain tissue was finely minced with scissors and digested in RPMI medium (Invitrogen) containing 2% FCS (Sigma Aldrich), 0.1 mg/ml DNase I (Sigma Aldrich), 200U/ml Collagenase I (GIBCO), 1mg/ml Dispase II (Roche) and 0.1mg/ml Liberase DL (Roche). Samples were incubated at 37°C for 30-45 minutes under agitation (900 rpm), with regular trituration. Microglia were identified as CD45^dim^ CD64^+^ CD11b^+^ Cx3cr1^+^.

### Flow cytometry and fluorescence-activated cell sorting (FACS)

Equal numbers of cells were stained with 1:1000 anti-CD16/32 (2.4G2; Biolegend) in 100µl of HBSS containing 1% BSA and 2mM EDTA (termed FACS buffer hereafter) for 15 minutes, to reduce non-specific antibody binding to receptors for IgG. Cells were subsequently stained with conjugated antibodies (Table S4) for 30 minutes at 4°C in the dark. Samples were washed with FACS buffer and centrifuged at 400G for 5 minutes at 4°C before resuspension in 300µl of FACS buffer. Single stain controls were prepared using OneComp Beads (ThermoFisher). Fluorescence minus one (FMO) controls were prepared using cells. Counting beads (ThermoFisher) were included to calculate absolute numbers. Dapi (Sigma Aldrich), SYTOX Green (ThermoFisher) or Zombie Fixable Viability Dye (Biolegend) was used as a dead cell marker. Samples were analyzed using an LSRII (BD Biosciences). Data was analyzed using FlowJo (V9 or V10).

### BrdU analysis

SMGs were harvested and single cells digested as above. Cells were stained using Zombie Fixable Viability Dye (Biolegend) and cell surface staining performed as above. Cells were fixed and washed using a FoxP3 intracellular staining kit (eBioscience) and incubated with 3 μg DNase I (Sigma Aldrich) and 1 ml 0.42M MgCl2 for 30 minutes at 37°C in the dark, before washing and staining with anti-BrdU antibody (Biolegend) and any other intracellular antibodies for 30 minutes at 37°C in the dark. Samples were washed and resuspended in FACS buffer before analysis, as above.

### Tissue processing for histology

SMGs were fixed for 6-8 hours in 4% paraformaldehyde (PFA; Thermo Scientific) at room temperature, with constant mixing, followed by 3 x washes with phosphate buffered saline (PBS; Merck). After fixation, SMGs were processed for the generation of frozen sections, by incubating in increasing concentrations of sucrose (10% and 30%) before embedding in OCT (Leica). 12 μm or 25 μm sections were cut using a cryostat (Leica) and stored at -20 °C.

### Immunofluorescent analysis

Whole-mount salivary gland and tissue section immunofluorescence analysis have been previously described (42, 87). In brief, tissue was fixed with 4% PFA if not previously fixed, and permeabilised with ice cold acetone/methanol (1:1) for 1 min. Tissue was blocked for 2 hours at room temperature with 5% BSA (Sigma Aldrich), 5% Donkey Serum (Merck) in 0.01% PBS-Tween-20 or donkey blocking buffer (0.5% saponin (Sigma Aldrich), 2% BSA, 1% fetal bovine serum (FBS; Sigma Aldrich) and 1% Donkey Serum for spectral imaging. Salivary glands were incubated with primary antibodies overnight at 4 °C. Antibodies are listed in Table S5. Antibodies were detected using donkey Cy2-, Cy3- or Cy5-conjugated secondary Fab fragment antibodies (Jackson Laboratories) and nuclei stained using Hoechst 33342 (1:1000, Sigma Aldrich), and mounted using Prolong Gold anti-fade mounting media or Prolong Diamond mounting media. Images were acquired on a Leica SP8 confocal microscope or a Zeiss LSM780 confocal microscope using the spectral unmixing mode. Fluorescent images were analyzed using NIH ImageJ software and Zen Blue.

T cell staining was achieved using a CD3 antibody (#100305, Biolegend, 1:100 in PBS/1% BSA, AB_312670), incubated at room temperature for 1 hour. The signal was amplified using rabbit anti-FITC conjugated to AF488 (#A11090, Life Technologies, 1:100 in PBS/1% BSA), incubated at room temperature for 1 hour, followed by further amplification with donkey anti-rabbit IgG FITC conjugated to AF488 (#A21206, Life Technologies, 1:100 in PBS/1% BSA) (88, 89). Co-staining for macrophage markers was then undertaken as described above.

### Histological cell counts

For immunofluorescent analysis, cells positively stained for markers were counted using ImageJ. 3 random fields of view per sample were taken on a Leica SP8 microscope at 40x magnification (Nyquist). Images were run through an ImageJ cell counting macro either as single images or, if the file was a z-stack, the middle image of the stack was used. Using the macro, images were split into individual channels and the appropriate channel was extracted. Positive cells, such as macrophages, were thresholded and counted according to their size using the “analyze particles” command. As a quality control an output file was saved where the counted macrophages were highlighted in green and could be manually checked/confirmed. The macro (Macro 1) is included in the Supplemental material. Fluorescence intensity was measured in Image J using the Threshold function.

### Nearest Neighbour analysis

Images were run through an ImageJ cell counting macro either as single images or, if the file was a z-stack, the middle image of the stack was used. Using the macro, images were split into individual channels and the appropriate channel (Hoescht) was extracted. Ductal nuclei were manually highlighted and a gradient was created. The channel containing macrophage staining was mapped on top and the distance between macrophages and nuclei was measured as the number of pixels. The macro (Macro 2) is included in the Supplemental material.

### Quantitative PCR analysis (qPCR)

RNA was isolated from whole tissue or sorted cells using the RNAqueous Micro Kit (Life Technologies). Total RNA samples were DNase-treated (Life Technologies) prior to cDNA synthesis (First Strand Synthesis Kit, ThermoFisher). SYBRgreen qPCR was performed using 5ng cDNA and primers designed using Primer3 and Beacon Designer software or described on PrimerBank (http://pga.mgh.harvard.edu/primerbank/). Primer sequences are listed in Table S6. Melt-curves and primer efficiency were determined as previously described (90). Gene expression was normalized to the housekeeping gene *Gapdh* and to the corresponding experimental control. Reactions were run in triplicate.

### Transcriptional profiling by scRNA-seq

Male or female C57BL/6 mice were irradiated (n=2 per group), as previously described and euthanised at 3 or 28 days post-irradiation. Non-irradiated control mice were not anaesthetized. Each SMG pair (approx. 160mg) was processed into a single cell suspension, as described for flow cytometry. Equal numbers of cells were stained with 1:1000 anti-CD16/32 (2.4G2; Biolegend) in FACS buffer for 15 minutes, to reduce non-specific antibody binding to receptors for IgG. Cells were subsequently stained with conjugated antibodies (Table S7) for 30 minutes at 4°C in the dark. Epithelial cells (EpCAM^+^), endothelial cells (CD31^+^) and macrophages (CD45^+^CD11b^+^F4/80^+^SiglecF^-^Ly6G^-^) were separated using a BD FACSAria II cell sorter and collected into HBSS containing 1% BSA. Cells were checked for number and viability with 1:1 Trypan Blue under a brightfield microscope. Cells were mixed in the following ratio to reach a final total of 10,000: epithelial cells at 45%, macrophages at 45%, endothelial cells at 10%. This was performed separately for samples collected from each individual mouse. Following this, samples for each experimental group were mixed together to create a mixture of cells from two individual biological replicates with a final total of 20,000 cells. The cells were processed for single cell barcoding using the 10x Chromium platform and a library prepared for each sample using the 10X Genomics single-cell RNA-seq 3’ V3.1 kit. Sequencing was performed on the NovaSeq 2×150bp platform to a depth of 350 million reads per sample.

### scRNA-seq pre-processing

Pre-processing of the data was performed using Seurat v4.0 according to the workflow suggested by the Satija lab (91). First, ambient RNA was identified by comparing the raw and filtered matrices and contaminant RNA estimated using the SoupX package (92). Adjusted matrices were then imputed into the R environment (v3.1) and analyzed using Seurat. Normalisation was performed using regularised negative binomial regression via the SCTransform function, including the removal of confounding variation arising from mitochondrial mapping percentage (91). Doublets were identified and removed using the “Doublet Finder” package via artificial next nearest neighbour analysis (93). Batch effect correction was assessed using the Harmony package (94). Identification of highly variable genes, next nearest neighbour, clustering functions and UMAP visualisation were all performed using the Seurat package. Marker genes per identified subpopulation were found using the findMarker function of the Seurat pipeline.

### Saliva Measurements

Saliva measurements were undertaken as previously described (95) prior to the start of the experiment (baseline) and on day 27 post-injury and calculated/presented as a percentage of the baseline measurement. Mice were injected with pilocarpine (resuspended in sterile water; 2mg/kg) (Sigma Aldrich) to stimulate saliva production. Mice were immediately restrained in a modified Greiner tube where the snout is exposed, and saliva was collected from the floor of the mouth into a sterile Eppendorf tube for 15 minutes via a blunt metal needle connected to a suction pump (Armo Line). Sample volumes were measured using a pipette.

### Statistical tests

Normal distribution was assessed using the D’Agostino-Pearson omnibus test. Data were analyzed for statistical significance using Student’s *t*-test (2 groups) or one-way ANOVA (multiple groups) with post-hoc testing performed using Tukey *Q* test (GraphPad Prism). For multiple testing we used a false discovery rate of 0.05. All graphs show the mean +standard deviation (SD), as indicated in the figure legends. Statistical tests used for each experiment are also indicated in all figure legends.

## Supplementary Material

Supplementary Material

## Data Availability

All data needed to evaluate the conclusions in the paper are present in the paper or the Supplementary Materials, and the RNA-seq data have been deposited in the European Molecular Biology Laboratory (EMBL) - European Bioinformatics Institute (EBI) public database ArrayExpress (https://www.ebi.ac.uk/biostudies/arrayexpress; accession code: E-MTAB-13374). Further information and requests for resources and reagents should be directed to and will be fulfilled by the lead contact, EE (Elaine.Emmerson@ed.ac.uk).

## One sentence summary

Long-lived macrophages in the submandibular salivary gland are necessary for regeneration after radiotherapy injury.
