# Supplementary material for: CSF1R-dependent macrophages in the salivary gland are essential for epithelial regeneration following radiation-induced injury
Source: Sci Immunol. Author manuscript; Available in PMC 2026 Jun 17. (PMC7619197; doi:10.1126/sciimmunol.add4374)
Supplement: Supplementary Material [file EMS214249-supplement-Supplementary_Material.pdf]

List of Supplementary Materials

Figure S1 – relates to Figure 1
Figure S2 – relates to Figure 2
Figure S3 – relates to Figure 3
Figure S4 – relates to Figure 4
Figure S5 – relates to Figure 4
Figure S6 – relates to Figure 5
Figure S7 – relates to Figure 6

Table S1: List of differentially expressed genes (DEG) between Cluster 0 and Cluster 1 – included in Supplementary Materials document
Table S2: List of differentially expressed genes (DEG) in subsets A-D – included in Supplementary Materials document
Table S3: Mouse strains used in this study, their source and relevant identifiers – included in Supplementary Materials document – included in Supplementary Materials document
Table S4: Antibodies used for flow cytometry – included in Supplementary Materials document
Table S5. Primary antibodies used for immunofluorescent staining – included in Supplementary Materials document
Table S6. Primer sequences used for qPCR — included in Supplementary Materials document
Table S7. Antibodies used for fluorescence activated cell sorting (FACS) – included in Supplementary Materials document

This manuscript has been accepted for publication in Science Immunology. This version has not undergone final editing. Please refer to the complete version of record at www.scienceimmunology.org. The manuscript may not be reproduced or used in any manner that does not fall within the fair use provisions of the Copyright Act without the prior, written permission of AAAS.

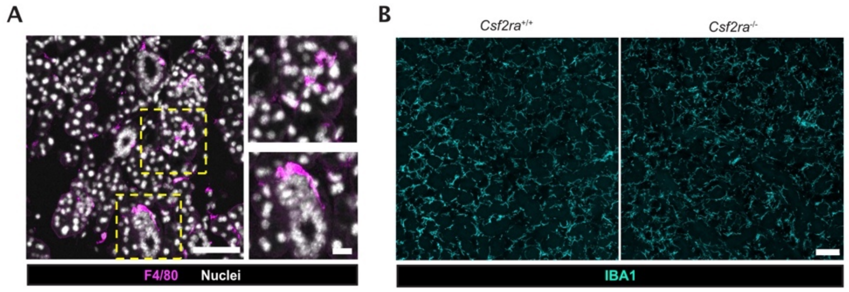

Figure S1 – relates to Figure 1

A. Representative expression of F4/80 in SMG tissue from unmanipulated adult C57BL/6J mice. Scale bars in large panel = 100μm, magnified insets = 10μm.

B. Representative expression of IBA1 in SMG tissue of *Csfr2a*$^{+/+}$ and *Csfr2a*$^{-/-}$ mice. Scale bar = 50μm.

This manuscript has been accepted for publication in Science Immunology. This version has not undergone final editing. Please refer to the complete version of record at www.scienceimmunology.org. The manuscript may not be reproduced or used in any manner that does not fall within the fair use provisions of the Copyright Act without the prior, written permission of AAAS.

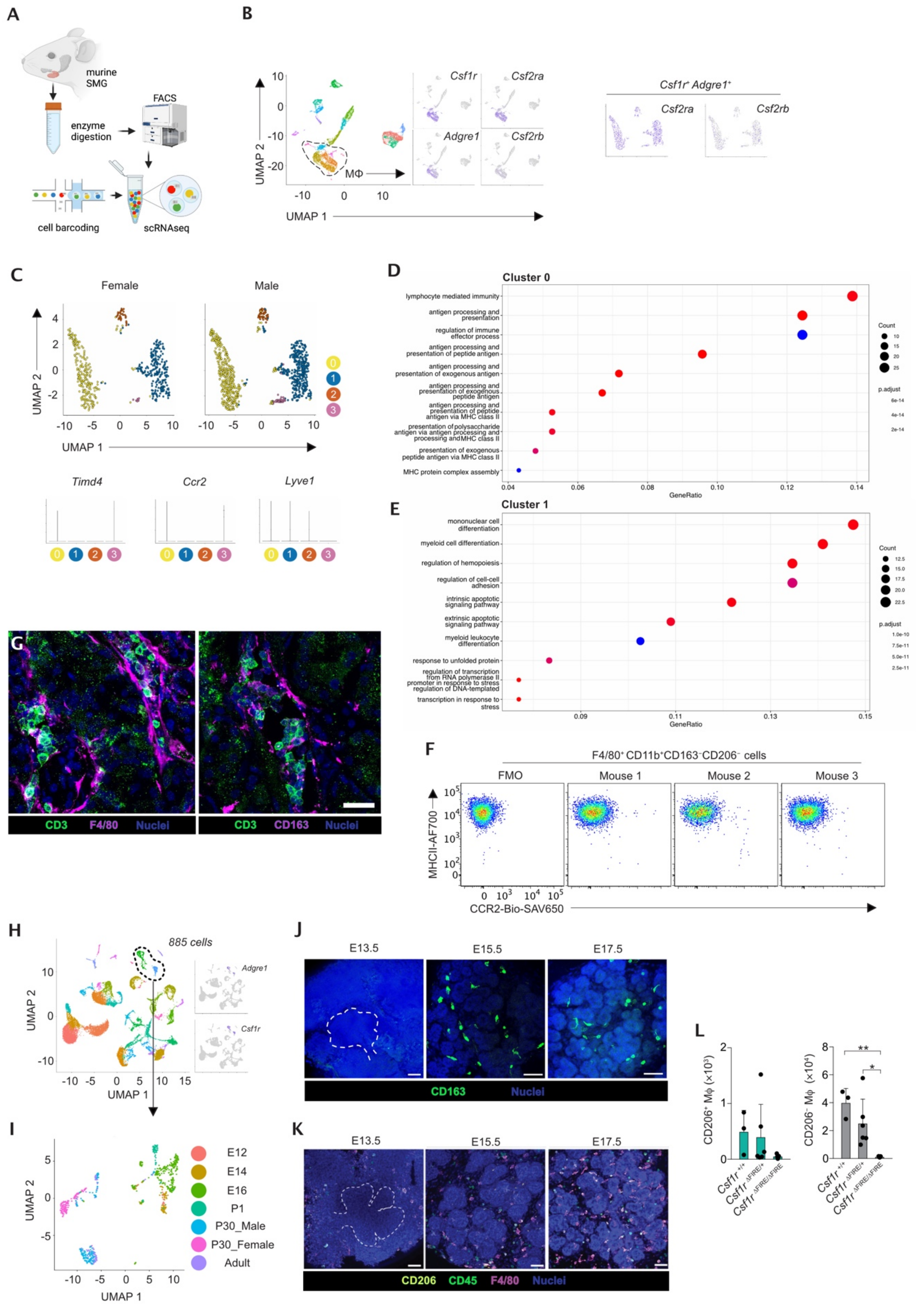

Figure S2 – relates to Figure 2

A. Schematic of scRNA-seq pipeline. Figure created in BioRender.com.

This manuscript has been accepted for publication in Science Immunology. This version has not undergone final editing. Please refer to the complete version of record at www.scienceimmunology.org. The manuscript may not be reproduced or used in any manner that does not fall within the fair use provisions of the Copyright Act without the prior, written permission of AAAS.

B. Feature plot showing expression of *Adgre1* and *Csf1r* to identify macrophages amongst epithelial and endothelial clusters, and insets to show expression of *Csf2ra* and *Csf2rb* on total cells and on re-clustered *Adgre1/Csf1r*$^{+}$ cells.

C. UMAP dimensionality reduction analysis of *Adgre1*$^{+}$*Csf1r*$^{+}$ cells from scRNA-seq of SMG of unmanipulated adult C57BL/6J mice (n=2, pooled), separated into female and male in order to show sexual dimorphism. Violin plots of *Timd4, Ccr2* and *Lyve1* expression by macrophage clusters 0-3.

D. Dotplot to show gene set enrichment analysis for Cluster 0.

E. Dotplot to show gene set enrichment analysis for Cluster 1.

F. Expression of CCR2 by F4/80^{+}CD11b^{+}CD163^{-}CD206^{-} SMG macrophages from the SMG of 3 unmanipulated adult C57BL/6J mice.

G. Representative expression of CD3, F4/80 and CD163 in SMG tissue of unmanipulated adult C57BL/6 mice. Scale bar = 25μm.

H. UMAP plot of gene expression from existing scRNA-seq atlas of murine SMG development *(32)* containing E12, E14, E16, P1, P30 (male and female) and adult (10 months of age) datasets from C3H mice with feature plots of *Adgre1* and *Csf1r* expression.

I. UMAP plot of gene expression of *Csf1r / Adgre1* expressing cells from (H) split by SMG developmental stage *(32)*.

J. Representative expression of CD163 in wholemount SMG tissue from unmanipulated embryonic (E13.5, E15.5, E17.5) C57BL/6J mice. Scale bars = 25mm. Dashed white line outlines SMG within the mesenchyme at E13.5.

K. Representative expression of CD45, F4/80 and CD206 in wholemount SMG tissue from unmanipulated embryonic (E13.5, E15.5, E17.5) C57BL/6J mice. Scale bars = 25μm. Dashed white line outlines SMG within the mesenchyme at E13.5.

L. Absolute number of CD206^{+} and CD206^{-} macrophages in the unmanipulated adult SMG of *Csf1r*$^{\Delta FIRE/\Delta FIRE}$ mice and their *Csf1r*$^{+/+}$ and *Csf1r*$^{\Delta FIRE/+}$ littermates. Data are from 3 (*Csf1r*$^{+/+}$), 5 (*Csf1r*$^{\Delta FIRE/+}$) or 5 (*Csf1r*$^{\Delta FIRE/\Delta FIRE}$) mice per group and are pooled from 2 independent experiments. *p<0.05, **p<0.01 (One-way ANOVA with post-hoc Tukey *Q* test) and error bars represent the SD.

This manuscript has been accepted for publication in Science Immunology. This version has not undergone final editing. Please refer to the complete version of record at www.scienceimmunology.org. The manuscript may not be reproduced or used in any manner that does not fall within the fair use provisions of the Copyright Act without the prior, written permission of AAAS.

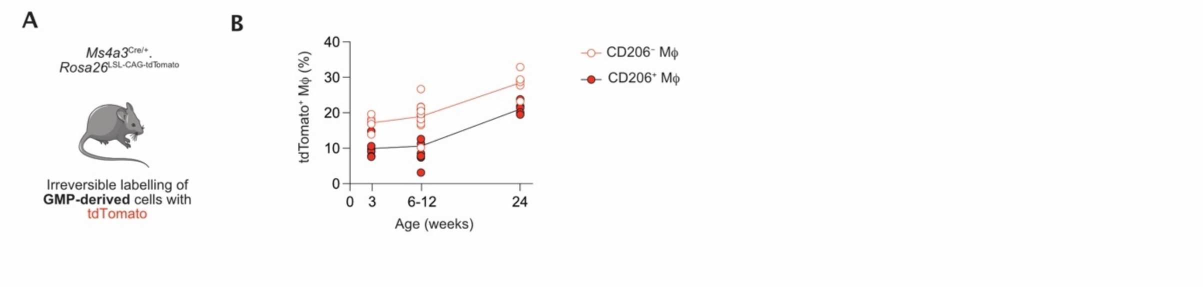

Figure S3 – relates to Figure 3

A. Description of *Ms4a3*$^{Cre/+}$.*Rosa26*$^{LSL\text{-}CAG\text{-}tdTomato}$ mice.

B. Graph shows the frequency of tdTomato^{+} cells amongst CD206-defined subsets obtained from *Ms4a3*$^{Cre/+}$.*Rosa26*$^{LSL\text{-}CAG\text{-}tdTomato}$ mice at the indicated ages. Data are from n=6 mice for 3- and 24 week time points, and n=10 for 6-12 week time points. Symbols represent individual mice.

This manuscript has been accepted for publication in Science Immunology. This version has not undergone final editing. Please refer to the complete version of record at www.scienceimmunology.org. The manuscript may not be reproduced or used in any manner that does not fall within the fair use provisions of the Copyright Act without the prior, written permission of AAAS.

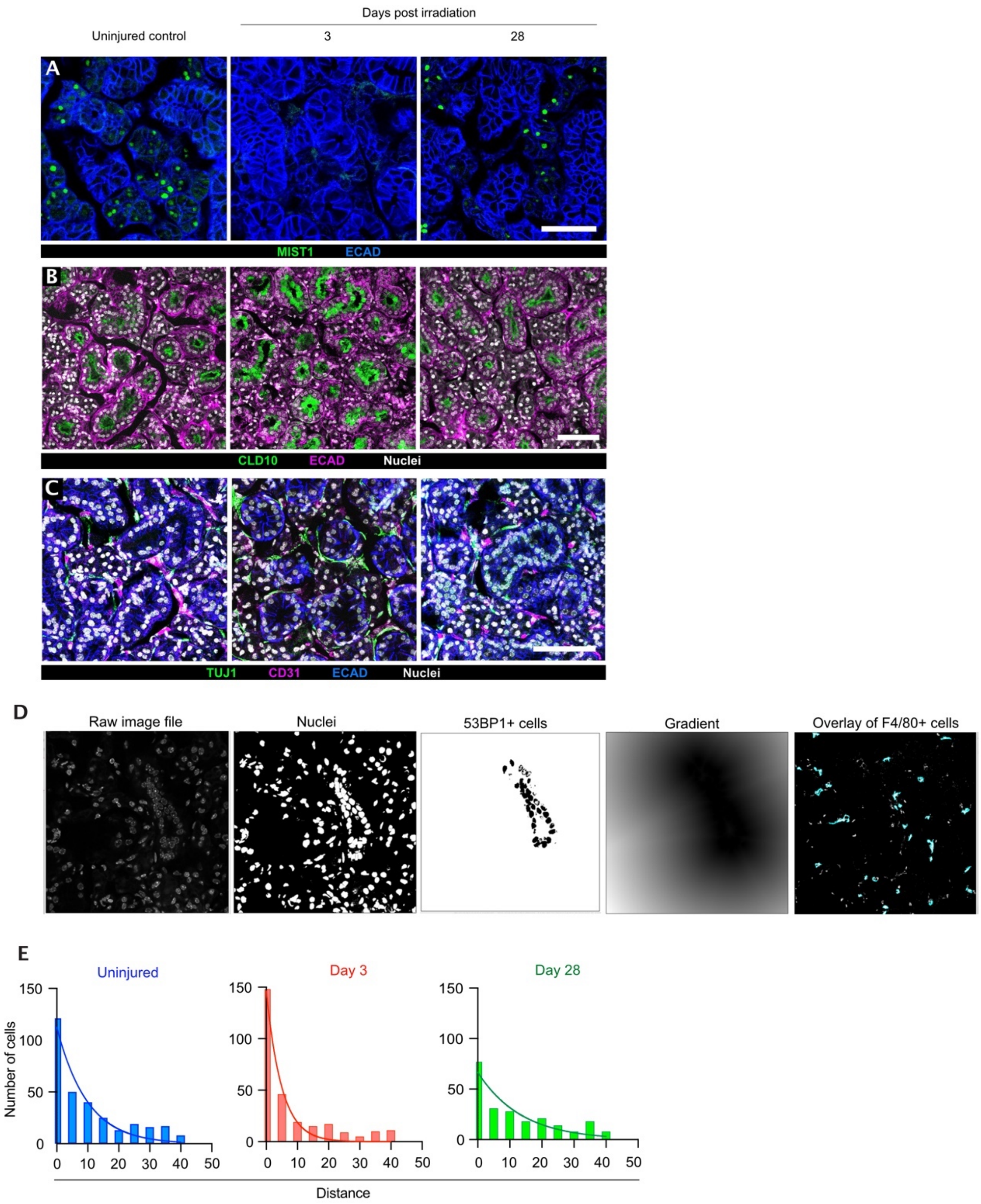

Figure S4 – relates to Figure 4

A-C. Representative expression of MIST1 and E-cadherin (ECAD) (**A**); claudin 10 (CLD10) and ECAD (**B**); beta III tubulin (TUJ1), CD31 and ECAD (**C**) in SMG of uninjured mice or mice irradiated 3 or 28 days earlier. Scale bars = 100μm.

D. Outline of the ImageJ image analysis pipeline for nearest neighbour analysis of macrophages and 53BP1+ cells. The software takes a raw image file, identifies nuclei, highlights those co-expressing 53BP1, creates a gradient from these dual positive cells and then overlays F4/80+ cells. The distance between F4/80+ cells and 53BP1+ cells can be measured using the gradient and pixel intensity.

E. Quantification of distances between macrophages and nuclei that contain 53BP1+ foci using nearest neighbour analysis of images of SMG of uninjured mice or mice irradiated 3 or 28 days earlier. Data obtained from three fields of view from non-sequential sections from 3 mice per timepoint. The line represents the line of best fit and is displayed in **Fig. 4F**.

This manuscript has been accepted for publication in Science Immunology. This version has not undergone final editing. Please refer to the complete version of record at www.scienceimmunology.org. The manuscript may not be reproduced or used in any manner that does not fall within the fair use provisions of the Copyright Act without the prior, written permission of AAAS.

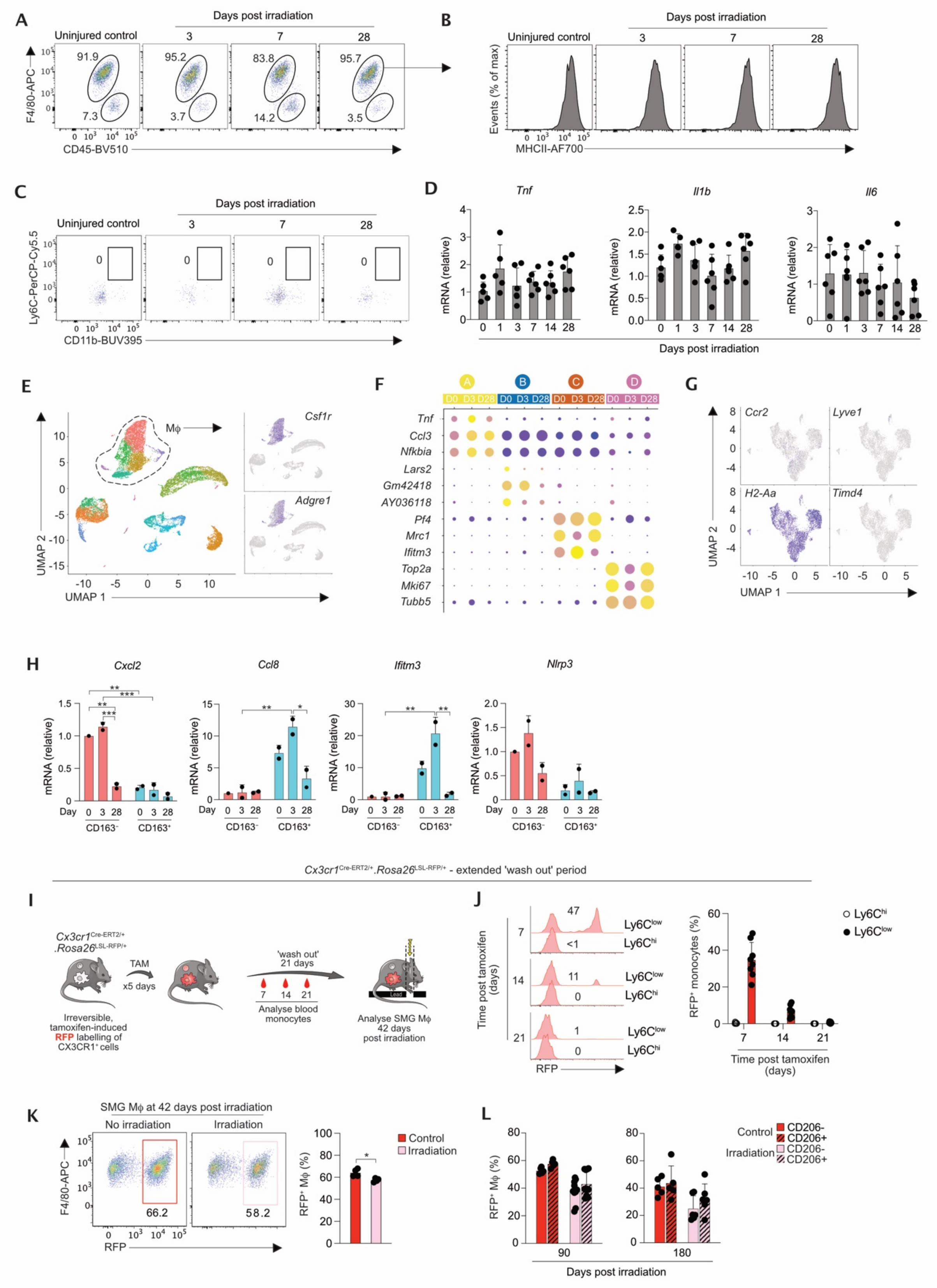

Figure S5 – relates to Figure 4

A. Representative expression of F4/80 and CD45 obtained from SMG of uninjured mice or mice irradiated 3, 7 or 28 days earlier.

B. Representative expression of MHCII by F4/80^{+}CD11b^{lo} macrophages obtained from SMG of uninjured mice or mice irradiated 3, 7 or 28 days earlier.

This manuscript has been accepted for publication in Science Immunology. This version has not undergone final editing. Please refer to the complete version of record at www.scienceimmunology.org. The manuscript may not be reproduced or used in any manner that does not fall within the fair use provisions of the Copyright Act without the prior, written permission of AAAS.

C. Representative expression of Ly6C and CD11b by F4/80^{-}CD45^{+} cells from SMG at 3, 7 or 28 days post radiation or from unmanipulated mice. Data are from one of two independent experiments performed.

D. qPCR analysis of *Tnf*, *Il1b* and *Il6* mRNA in total SMG tissue at the indicated time points following radiation induced injury. Data are normalized to mRNA levels in unmanipulated naïve (D0) SMG tissue. Data are from 5-6 mice pooled from 2 independent experiments and error bars represent the SD.

E. UMAP dimensionality reduction analysis of scRNA-Seq data from 14,855 cells containing macrophages, epithelia and endothelia from SMG of unmanipulated mice or mice irradiated 3 or 28 days earlier. Feature plots showing expression of *Adgre1* (encoding F4/80) and *Csf1r* to identify macrophages (Mϕ).

F. Bubble plot showing expression of selected genes by macrophage clusters from unmanipulated (D0) or at day 3 (D3) or day 28 (D28) following radiation treatment.

G. Feature plots showing expression of *Ccr2, Lyve1, H2-Aa and Timd4* amongst macrophage subsets (as depicted in **Fig. 4G**).

H. qPCR analysis of *Cxcl2, Ccl8, Ifitm3* and *Nlrp3* in sorted CD163^{-} and CD163^{+} macrophages from SMG of unmanipulated mice or mice irradiated 3 or 28 days earlier. Data are normalized to mRNA levels in CD163^{-} cells from SMG exposed to 0Gy. Data are from 4 mice per group, sorted into two samples per group. *p<0.05, **p<0.01, ***p<0.001 (One-way ANOVA followed by Tukey *Q* post-hoc test) and error bars represent the SD.

I. Experimental scheme for fate mapping CX3CR1^{+} cells in adult *Cx3cr1*$^{\text{Cre-ERT2/+}}$.*Rosa26*$^{\text{LSL-RFP}}$ mice with an extended wash-out period.

J. Representative RFP expression by Ly6C-defined blood monocytes at the indicated timepoints following tamoxifen cessation. Graph shows the frequency of RFP^{+} monocytes at the indicated timepoints. Data are from 8 mice and error bars represent the SD.

K. Representative RFP expression amongst SMG macrophages 42 days post-tamoxifen. Graph shows the frequency of RFP+ macrophages with and without irradiation. Data are from 4 mice per group. *p<0.05 (Student's *t* test) and error bars represent the SD.

L. Frequency of RFP^{+} cells amongst F4/80^{+}CD11b^{lo}CD206^{+}CD163^{+} versus F4/80^{+}CD11b^{lo}CD206^{-}CD163^{-} SMG macrophages obtained from *Cx3cr1*$^{\text{Cre-ERT2/+}}$.*Rosa26*$^{\text{LSL-RFP}}$ mice administered tamoxifen by oral gavage for 5 days and analyzed at the indicated time points following targeted radiation. Data are from 5-11 mice pooled from 3 independent experiments. Error bars represent the SD.

This manuscript has been accepted for publication in Science Immunology. This version has not undergone final editing. Please refer to the complete version of record at www.scienceimmunology.org. The manuscript may not be reproduced or used in any manner that does not fall within the fair use provisions of the Copyright Act without the prior, written permission of AAAS.

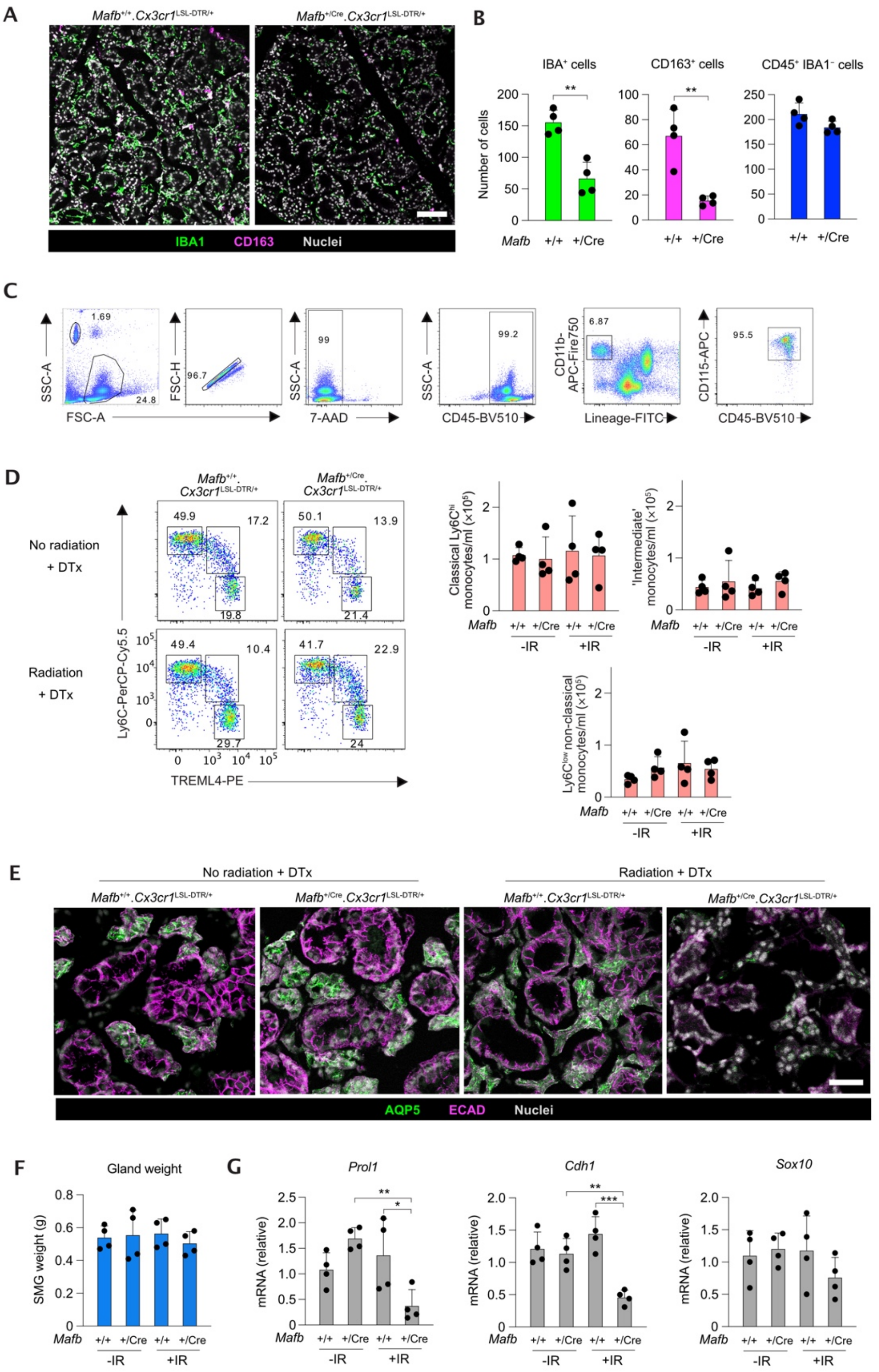

Figure S6 – relates to Figure 5

A. Representative expression of IBA1 and CD163 in SMG of uninjured *Mafb*$^{Cre/+}$.*Cx3cr1*$^{LSL\text{-}DTR/+}$ mice or *Mafb*$^{+/+}$.*Cx3cr1*$^{LSL\text{-}DTR/+}$ littermates administered diphtheria toxin (DTx) or saline every 2 days for 10 days. Scale bar = 100μm.

B. Enumeration of IBA1^{+} cells, CD163^{+} cells and CD45^{+}IBA1^{-} cells (i.e. other leukocytes) in *Mafb*$^{Cre/+}$.*Cx3cr1*$^{LSL\text{-}DTR/+}$ mice or *Mafb*$^{+/+}$.*Cx3cr1*$^{LSL\text{-}DTR/+}$ littermates exposed to 0Gy or 10Gy irradiation and administered diphtheria toxin (DTx) or saline from day 17 onwards, every 2 days and analyzed 28 days after irradiation. Data obtained from three fields of view from non-sequential sections from 4 mice per group. **p<0.01 (One-way ANOVA with Tukey *Q* post-hoc testing).

[#] This manuscript has been accepted for publication in Science Immunology. This version has not undergone final editing. Please refer to the complete version of record at www.scienceimmunology.org. The manuscript may not be reproduced or used in any manner that does not fall within the fair use provisions of the Copyright Act without the prior, written permission of AAAS.

C. Gating strategy for the identification of blood monocytes.

D. Representative expression of Ly6C and TREML4 by CD115$^+$ monocytes in *Mafb*$^{Cre/+}$.*Cx3cr1*$^{LSL\text{-}DTR/+}$ mice or *Mafb*$^{+/+}$.*Cx3cr1*$^{LSL\text{-}DTR/+}$ littermate controls with or without 10Gy irradiation. Graphs show the absolute number of monocyte subsets in the indicated groups. Data are from 4 mice per group.

E. Additional immunofluorescent images of SMG stained for AQP5 and ECAD in *Mafb*$^{Cre/+}$.*Cx3cr1*$^{LSL\text{-}DTR/+}$ mice or *Mafb*$^{+/+}$.*Cx3cr1*$^{LSL\text{-}DTR/+}$ littermates exposed to 0Gy or 10Gy irradiation and administered diphtheria toxin (DTx) or saline from day 17 onwards, every 2 days and analyzed 28 days after irradiation. Scale bar = 25μm.

F. Quantification of the weight of a single SMG from *Mafb*$^{Cre/+}$.*Cx3cr1*$^{LSL\text{-}DTR/+}$ mice or *Mafb*$^{+/+}$.*Cx3cr1*$^{LSL\text{-}DTR/+}$ littermates exposed to 0Gy or 10Gy irradiation and administered diphtheria toxin (DTx) or saline from day 17 onwards, every 2 days and analyzed 28 days after irradiation. Data represent 1 SMG per mouse and are from 4 mice per group.

G. qPCR analysis of *Prol1* (Mucin 10), *Cdh1 and Sox10* mRNA in total SMG tissue in *Mafb*$^{Cre/+}$.*Cx3cr1*$^{LSL\text{-}DTR/+}$ mice or *Mafb*$^{+/+}$.*Cx3cr1*$^{LSL\text{-}DTR/+}$ littermates exposed to 0Gy or 10Gy irradiation and administered diphtheria toxin (DTx) or saline from day 17 onwards, every 2 days and analyzed 28 days after irradiation. Data are normalized to mRNA levels in SMG tissue from *Mafb*$^{+/+}$.*Cx3cr1*$^{LSL\text{-}DTR/+}$ littermates exposed to 0Gy. Data are from 4 mice per group. *p<0.05, **p<0.01, ***p<0.001 (One-way ANOVA followed by Tukey *Q* post-hoc test).

This manuscript has been accepted for publication in Science Immunology. This version has not undergone final editing. Please refer to the complete version of record at www.scienceimmunology.org. The manuscript may not be reproduced or used in any manner that does not fall within the fair use provisions of the Copyright Act without the prior, written permission of AAAS.

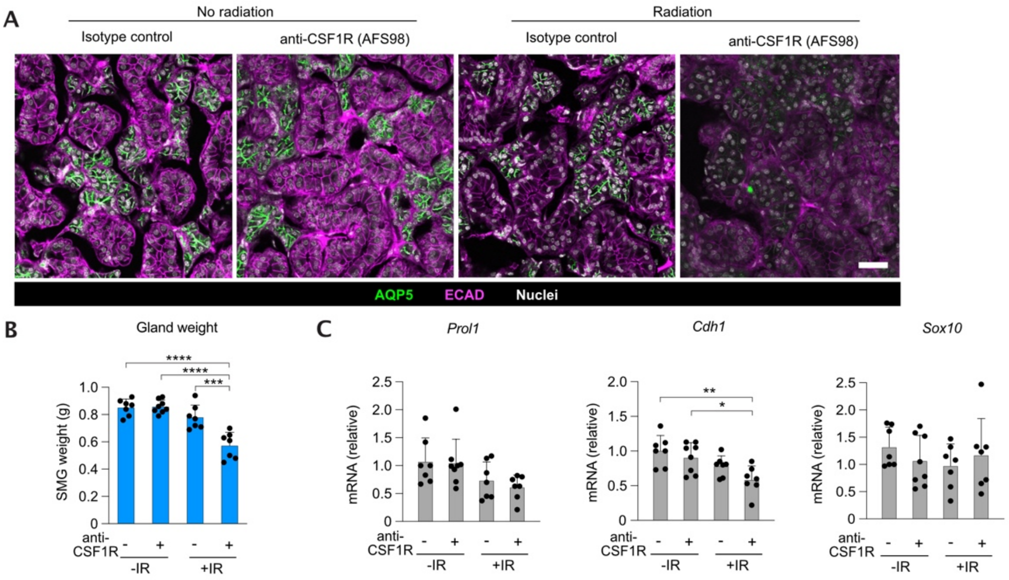

Figure S7 – relates to Figure 6

A. Additional immunofluorescent images of SMG stained for AQP5 and ECAD in C57BL/6 mice exposed to 0Gy or 10Gy irradiation before being administered anti-CSF1R (AFS98) or isotype control and analyzed 28 days after irradiation. Scale bar = 25μm.

B. Quantification of the weight of a single SMG from C57BL/6 mice exposed to 0Gy or 10Gy irradiation before being administered anti-CSF1R (AFS98) or isotype control and analyzed 28 days after irradiation. Data represent 1 SMG per mouse and are from 7-8 mice per group. ***p<0.001, ****p<0.0001 (One-way ANOVA followed by Tukey *Q* post-hoc test).

C. qPCR analysis of *Prol1* (Mucin 10), *Cdh1 and Sox10* mRNA in total SMG tissue from C57BL/6 mice exposed to 0Gy or 10Gy irradiation before being administered anti-CSF1R (AFS98) or isotype control and analyzed 28 days after irradiation. Data are normalized to mRNA levels in SMG tissue from mice administered isotype control and exposed to 0Gy. Data are from 7-8 mice per group. *p<0.05, **p<0.01 (One-way ANOVA followed by Tukey *Q* post-hoc test).

This manuscript has been accepted for publication in Science Immunology. This version has not undergone final editing. Please refer to the complete version of record at www.scienceimmunology.org. The manuscript may not be reproduced or used in any manner that does not fall within the fair use provisions of the Copyright Act without the prior, written permission of AAAS.

Table S1: List of differentially expressed genes (DEG) between Cluster 0 and Cluster 1

	p_val	avg_log2FC	pct.1	pct.2	p_val_adj
Nfkbia	6.8E-163	2.917051	0.928	0.308	7.8E-159
Cd83	1.1E-156	2.870531	0.891	0.246	1.3E-152
Atf3	1.9E-152	3.201957	0.914	0.337	2.2E-148
Ccl4	9.3E-139	2.89825	0.961	0.566	1.1E-134
Hspa8	3.6E-136	2.088057	0.971	0.692	4.1E-132
Zfp36	4.6E-135	2.424868	0.905	0.358	5.2E-131
Ccl3	1.3E-130	2.98736	0.907	0.392	1.5E-126
Tnfaip3	3.1E-130	1.85266	0.735	0.066	3.6E-126
Junb	2.5E-128	2.432195	0.912	0.441	2.9E-124
Hspa1a	7.4E-126	2.495079	0.925	0.59	8.4E-122
Nfkbiz	3.1E-124	1.973946	0.834	0.245	3.6E-120
Ubc	1.5E-123	2.389374	0.912	0.455	1.7E-119
Hsp90aa1	3.7E-123	2.905585	0.89	0.404	4.2E-119
Hspa1b	1.9E-113	2.082154	0.918	0.517	2.1E-109
Ccrl2	2.8E-110	1.853533	0.739	0.136	3.2E-106
Ifrd1	4.4E-109	2.04431	0.626	0.036	5E-105
Tnf	9.6E-104	2.339341	0.671	0.094	1.1E-99
Ctss	3.18E-96	-0.95607	0.984	1	3.63E-92
Il1b	3.48E-96	2.515623	0.803	0.332	3.98E-92
Hsph1	4.16E-95	1.920327	0.648	0.096	4.75E-91
Egr1	1.94E-94	2.212281	0.641	0.099	2.22E-90
Ppp1r15a	4.62E-94	1.764147	0.669	0.11	5.28E-90
Mcl1	3.77E-92	1.483892	0.914	0.554	4.3E-88
Hsp90ab1	1.5E-88	1.659149	0.964	0.841	1.72E-84
Pim1	2.94E-88	1.661133	0.653	0.131	3.36E-84
Tent5c	1.33E-85	1.213564	0.782	0.261	1.52E-81
Rgs1	2.39E-85	1.688423	0.934	0.608	2.73E-81
Dnajb1	5.08E-85	2.556463	0.73	0.259	5.8E-81
Cxcl2	4.47E-84	3.664485	0.64	0.162	5.1E-80
Socs3	4.57E-83	1.716416	0.61	0.1	5.22E-79
C1qa	1.76E-78	-0.8584	0.993	0.997	2.01E-74
Nfe2l2	7.22E-78	1.25602	0.839	0.481	8.25E-74
C1qc	9.49E-78	-0.78334	0.984	0.998	1.08E-73
Il1a	1.18E-77	1.853267	0.626	0.162	1.35E-73
Klf6	2.38E-77	1.604177	0.854	0.47	2.72E-73
Dusp1	3.38E-77	1.484945	0.875	0.41	3.87E-73
Ubb	2.21E-76	1.811827	0.915	0.676	2.52E-72
Hexb	4.39E-75	-0.9738	0.924	0.994	5.02E-71
Tlr2	5.86E-74	1.34792	0.766	0.36	6.69E-70
Itm2b	1.3E-73	-1.00509	0.888	0.994	1.48E-69

[#] This manuscript has been accepted for publication in Science Immunology. This version has not undergone final editing. Please refer to the complete version of record at www.scienceimmunology.org. The manuscript may not be reproduced or used in any manner that does not fall within the fair use provisions of the Copyright Act without the prior, written permission of AAAS.

Jun	1.12E-72	2.013438	0.834	0.524	1.29E-68
Ier5	2.7E-70	1.193986	0.867	0.48	3.09E-66
Kdm6b	1.03E-69	1.151292	0.484	0.049	1.17E-65
Fos	1.41E-66	1.596723	0.832	0.46	1.61E-62
Sqstm1	1.79E-65	1.320858	0.601	0.165	2.04E-61
Rasgef1b	4.65E-65	0.999105	0.473	0.057	5.32E-61
C1qb	1.32E-64	-0.74006	0.99	0.998	1.51E-60
Zfand5	4.14E-64	1.143422	0.727	0.303	4.73E-60
H3f3b	1.08E-61	1.432387	0.947	0.797	1.23E-57
Cyba	1.06E-60	-0.90191	0.779	0.968	1.21E-56
Fosb	4.56E-60	1.453001	0.539	0.115	5.21E-56
Ccnl1	2.84E-58	0.949053	0.783	0.42	3.24E-54
H2-Aa	4.14E-58	-0.6324	0.997	1	4.73E-54
Tmem176a	7.24E-57	-0.95269	0.654	0.909	8.28E-53
Skil	7.48E-56	1.016885	0.844	0.528	8.55E-52
Rel	2.13E-55	1.062014	0.663	0.267	2.44E-51
Dusp2	3.56E-55	1.318946	0.393	0.032	4.06E-51
Hspe1	6.36E-55	1.30032	0.812	0.562	7.26E-51
Lilr4b	1.19E-54	1.195172	0.604	0.203	1.36E-50
Gadd45b	6.48E-54	1.52824	0.349	0.013	7.4E-50
Man2b1	1.89E-53	-0.85057	0.603	0.865	2.16E-49
Nr4a1	1.38E-51	1.073207	0.37	0.031	1.58E-47
Cd81	1.8E-51	-0.89982	0.739	0.937	2.05E-47
H2-Ab1	1.4E-50	-0.65843	0.993	1	1.6E-46
Tyrobp	3.58E-50	-0.71518	0.976	0.997	4.09E-46
Clic4	7.66E-48	1.011376	0.603	0.23	8.75E-44
Tgif1	9.25E-48	0.854427	0.539	0.175	1.06E-43
Cdkn1a	8.74E-47	1.096857	0.442	0.094	9.99E-43
Sdc4	1.5E-46	1.303855	0.453	0.107	1.72E-42
Psap	1.97E-46	-0.71829	0.938	0.985	2.25E-42
Cxcl10	1.14E-44	2.325941	0.448	0.11	1.3E-40
H2-Eb1	5.86E-44	-0.49413	0.997	1	6.7E-40
Bcl2a1d	6.92E-44	1.084737	0.588	0.259	7.9E-40
Tmem176b	9.02E-44	-0.72518	0.845	0.953	1.03E-39
Fcer1g	2.94E-42	-0.64137	0.976	0.997	3.36E-38
Dnaja1	4.69E-42	1.351894	0.653	0.324	5.36E-38
Bag3	2.98E-41	0.866798	0.347	0.049	3.41E-37
Cebpb	3.39E-41	1.126762	0.524	0.183	3.87E-37
H2-D1	4.44E-41	-0.73174	0.858	0.959	5.07E-37
Laptm5	6.64E-41	-0.58792	0.934	0.985	7.58E-37
Csrnp1	1.44E-40	0.59558	0.28	0.013	1.65E-36
Rab20	1.74E-40	0.734637	0.473	0.149	1.99E-36
Bcl2a1b	3.02E-40	1.057648	0.894	0.76	3.45E-36
Sowahc	3.25E-40	0.898361	0.531	0.186	3.71E-36

This manuscript has been accepted for publication in Science Immunology. This version has not undergone final editing. Please refer to the complete version of record at www.scienceimmunology.org. The manuscript may not be reproduced or used in any manner that does not fall within the fair use provisions of the Copyright Act without the prior, written permission of AAAS.

Lilrb4a	7.44E-40	0.92142	0.785	0.511	8.5E-36
Srgn	3.65E-39	0.957968	0.879	0.728	4.17E-35
Cst3	4.66E-38	-0.57764	0.986	0.998	5.33E-34
Plau	6.66E-38	0.95398	0.478	0.164	7.61E-34
Btg2	2.9E-37	1.268967	0.65	0.347	3.31E-33
Gna13	3.1E-37	0.722679	0.512	0.188	3.55E-33
Tnfsf9	5.04E-36	1.079338	0.263	0.018	5.76E-32
Phlda1	9.42E-34	1.363251	0.326	0.06	1.08E-29
Id2	1.08E-33	1.174677	0.403	0.113	1.23E-29
Cd14	3.33E-33	1.410322	0.791	0.635	3.8E-29
Ctsz	4.37E-33	-0.633	0.881	0.972	4.99E-29
Hmox1	1.31E-32	1.455409	0.538	0.263	1.49E-28
Cx3cr1	1.25E-31	-0.63868	0.846	0.958	1.43E-27
Rilpl2	2.06E-31	0.630064	0.422	0.139	2.35E-27
Basp1	2.91E-31	0.749821	0.917	0.817	3.33E-27
Jund	3.11E-31	1.21199	0.653	0.374	3.55E-27
Ctsh	4.2E-31	-0.58094	0.819	0.948	4.8E-27
Cxcr4	5.1E-31	0.646599	0.35	0.089	5.83E-27
Tmem86a	6.99E-31	-0.53568	0.179	0.462	7.99E-27
Tagap	1.04E-30	0.735553	0.485	0.198	1.19E-26
Grn	1.73E-29	-0.68737	0.379	0.637	1.97E-25
H2-DMa	2.8E-29	-0.53873	0.783	0.929	3.2E-25
Nfkbid	6.13E-29	0.486521	0.319	0.071	7E-25
Ms4a7	1.43E-28	-0.678	0.836	0.942	1.63E-24
Unc93b1	2.47E-28	-0.53787	0.801	0.911	2.82E-24
Nlrp3	4.75E-28	0.717197	0.293	0.063	5.43E-24
Lamp1	8.06E-28	-0.57189	0.578	0.781	9.21E-24
Tiparp	6.97E-27	0.565343	0.392	0.133	7.96E-23
Erp29	1.74E-26	-0.5833	0.415	0.66	1.98E-22
Apoe	6.14E-26	-0.65807	0.941	0.987	7.02E-22
Cd63	1.05E-25	-0.68166	0.568	0.757	1.2E-21
Coq10b	1.28E-25	0.55382	0.461	0.193	1.46E-21
Sdcbp	1.66E-25	0.652332	0.72	0.488	1.9E-21
Plekho2	2.59E-25	0.472472	0.397	0.146	2.96E-21
Lgmn	2.85E-25	-0.54718	0.815	0.927	3.25E-21
Cd74	3.86E-25	-0.41598	0.999	1	4.41E-21
Cd52	5.18E-25	-0.49411	0.966	0.99	5.92E-21
Aif1	6.1E-25	-0.53326	0.849	0.948	6.97E-21
Ndel1	8.34E-25	0.425657	0.329	0.097	9.53E-21
Btg1	5.05E-24	0.755377	0.768	0.585	5.78E-20
Icam1	1.42E-23	0.784219	0.286	0.073	1.62E-19
Ptger4	1.96E-23	0.511389	0.337	0.113	2.24E-19
Tank	1.65E-22	0.409074	0.339	0.113	1.88E-18
Ckb	2.42E-22	-0.55299	0.489	0.708	2.77E-18

This manuscript has been accepted for publication in Science Immunology. This version has not undergone final editing. Please refer to the complete version of record at www.scienceimmunology.org. The manuscript may not be reproduced or used in any manner that does not fall within the fair use provisions of the Copyright Act without the prior, written permission of AAAS.

Dennd4a	2.51E-22	0.600574	0.362	0.136	2.87E-18
Lst1	3.9E-22	-0.52785	0.679	0.846	4.46E-18
Clic1	6.63E-22	-0.48344	0.71	0.865	7.58E-18
H2-DMb1	7.89E-22	-0.55598	0.742	0.867	9.01E-18
Tmem119	1.16E-21	-0.52813	0.409	0.645	1.33E-17
Pfn1	1.27E-21	-0.57248	0.782	0.898	1.46E-17
Tmem59	1.62E-21	-0.46629	0.178	0.407	1.85E-17
Rnf19b	2.24E-21	0.378109	0.261	0.065	2.56E-17
Pdia3	4.74E-21	-0.51634	0.568	0.773	5.42E-17
Arl5c	9.32E-21	0.691744	0.264	0.075	1.06E-16
Pnrc1	1.1E-20	0.582083	0.488	0.261	1.26E-16
Picalm	1.65E-20	0.557341	0.772	0.574	1.89E-16
Trf	1.78E-20	-0.57812	0.403	0.609	2.04E-16
Mrps6	4E-20	0.616004	0.3	0.099	4.57E-16
Tspo	7.23E-20	-0.57542	0.254	0.464	8.26E-16
Hexa	8.26E-20	-0.5031	0.539	0.718	9.44E-16
Pld4	1.04E-19	-0.47512	0.519	0.708	1.19E-15
Psmb8	1.06E-19	-0.51669	0.473	0.665	1.21E-15
Litaf	2.26E-19	0.417202	0.4	0.177	2.58E-15
Atox1	3.19E-19	-0.49634	0.633	0.804	3.65E-15
Vsir	3.88E-19	-0.46301	0.334	0.556	4.44E-15
Tmem50a	3.9E-19	-0.45997	0.344	0.561	4.45E-15
Gusb	5.32E-19	-0.44149	0.296	0.519	6.08E-15
Herpud1	6.01E-19	0.582267	0.377	0.17	6.87E-15
P2ry6	7.3E-19	-0.45992	0.341	0.558	8.35E-15
Fth1	8.73E-19	0.429141	0.997	0.994	9.98E-15
Creg1	1.16E-18	-0.4744	0.323	0.53	1.32E-14
Tnfaip8l2	1.74E-18	-0.42243	0.208	0.418	1.99E-14
Plek	2.01E-18	0.606273	0.549	0.348	2.29E-14
Hsp90b1	3.01E-18	-0.48554	0.572	0.762	3.44E-14
Prdx1	3.67E-18	-0.5572	0.494	0.679	4.19E-14
F11r	4.56E-18	-0.46178	0.395	0.59	5.21E-14
Psmb10	5.01E-18	-0.41061	0.187	0.387	5.72E-14
B2m	1.17E-17	-0.34884	0.997	0.997	1.33E-13
Txnip	1.96E-17	-0.39165	0.281	0.512	2.23E-13
Fcgr3	2E-17	-0.42718	0.512	0.694	2.28E-13
Calr	2.08E-17	-0.55461	0.449	0.652	2.38E-13
Gnai2	2.57E-17	-0.40836	0.722	0.84	2.94E-13
Ahsa1	2.92E-17	0.403754	0.43	0.219	3.33E-13
Cd69	3.51E-17	0.647715	0.287	0.102	4.02E-13
Ly86	3.55E-17	-0.37883	0.907	0.959	4.05E-13
Cxcl16	4.15E-17	0.506771	0.905	0.856	4.74E-13
Spag9	4.75E-17	0.469137	0.422	0.216	5.43E-13
Metrnl	5.71E-17	0.42208	0.383	0.183	6.52E-13

This manuscript has been accepted for publication in Science Immunology. This version has not undergone final editing. Please refer to the complete version of record at www.scienceimmunology.org. The manuscript may not be reproduced or used in any manner that does not fall within the fair use provisions of the Copyright Act without the prior, written permission of AAAS.

Slamf9	8.2E-17	-0.36896	0.653	0.814	9.37E-13
Csf1r	1.06E-16	-0.42128	0.871	0.951	1.22E-12
Slc38a2	1.16E-16	0.493863	0.362	0.167	1.33E-12
Itm2c	1.22E-16	-0.45409	0.352	0.538	1.4E-12
Ms4a6b	1.53E-16	-0.48335	0.407	0.598	1.74E-12
Sh3bgrl3	3.19E-16	-0.45429	0.819	0.908	3.65E-12
Ly6e	3.73E-16	-0.62262	0.3	0.501	4.27E-12
Coro1a	7.13E-16	-0.4636	0.768	0.856	8.15E-12
Serinc3	8.49E-16	-0.38847	0.875	0.927	9.7E-12
Hmgb1	9.75E-16	-0.42417	0.409	0.611	1.11E-11
Tsc22d4	1.02E-15	-0.43489	0.258	0.444	1.16E-11
Ifngr1	1.23E-15	-0.45241	0.429	0.613	1.41E-11
Dyrk2	1.31E-15	0.322307	0.258	0.091	1.49E-11
Serf2	1.49E-15	-0.5292	0.405	0.605	1.7E-11
Zfp622	2.02E-15	0.328448	0.261	0.094	2.31E-11
Psme1	2.24E-15	-0.40715	0.601	0.765	2.56E-11
Ctsc	2.31E-15	-0.33243	0.888	0.966	2.64E-11
Cmtm7	3.17E-15	-0.421	0.33	0.514	3.62E-11
Laptm4a	3.22E-15	-0.44742	0.333	0.527	3.68E-11
Olfml3	3.31E-15	-0.50401	0.339	0.507	3.78E-11
Tmed9	3.65E-15	-0.36915	0.188	0.371	4.17E-11
Itgb5	4.26E-15	-0.42907	0.555	0.716	4.87E-11
Cd68	4.94E-15	-0.41879	0.768	0.844	5.64E-11
Hspd1	6.92E-15	0.583505	0.387	0.206	7.9E-11
Lyz2	6.93E-15	-0.71441	0.669	0.828	7.92E-11
Tmbim6	8.49E-15	-0.45046	0.311	0.491	9.71E-11
Plbd1	8.71E-15	-0.39252	0.492	0.663	9.95E-11
Selenof	9.93E-15	-0.34805	0.176	0.355	1.13E-10
Ppib	1.13E-14	-0.41289	0.373	0.558	1.29E-10
Fcgr1	1.14E-14	-0.42303	0.426	0.613	1.3E-10
H2-K1	1.2E-14	-0.50186	0.7	0.817	1.37E-10
Timp2	1.28E-14	-0.40572	0.349	0.535	1.46E-10
Bst2	1.29E-14	-0.50727	0.798	0.88	1.48E-10
Stab1	1.88E-14	-0.4015	0.291	0.48	2.14E-10
Irf1	2.05E-14	0.550967	0.265	0.105	2.34E-10
Kctd12	2.61E-14	0.610298	0.717	0.561	2.99E-10
H2-T23	2.84E-14	-0.47553	0.39	0.554	3.25E-10
Fxyd5	3.62E-14	-0.42072	0.657	0.809	4.14E-10
Atf4	5.61E-14	0.312742	0.261	0.1	6.41E-10
Srsf9	6.28E-14	-0.31639	0.228	0.413	7.17E-10
Bcl2a1a	1.03E-13	0.554066	0.505	0.331	1.17E-09
Bach1	1.75E-13	0.402197	0.356	0.182	1.99E-09
Gpx1	2.28E-13	-0.41606	0.386	0.569	2.61E-09
Nup98	3.05E-13	0.25508	0.265	0.109	3.49E-09

This manuscript has been accepted for publication in Science Immunology. This version has not undergone final editing. Please refer to the complete version of record at www.scienceimmunology.org. The manuscript may not be reproduced or used in any manner that does not fall within the fair use provisions of the Copyright Act without the prior, written permission of AAAS.

Samsn1	3.3E-13	0.314304	0.311	0.146	3.77E-09
Iqgap1	3.91E-13	0.474829	0.756	0.619	4.47E-09
Ptms	4.9E-13	-0.43472	0.496	0.639	5.6E-09
Ppfia4	5.92E-13	-0.37029	0.402	0.562	6.76E-09
N4bp1	6E-13	0.30112	0.26	0.105	6.86E-09
Trem2	6.65E-13	-0.52381	0.397	0.546	7.6E-09
Efhd2	7.87E-13	0.400783	0.746	0.601	9E-09
Gm6377	9.83E-13	0.334689	0.257	0.104	1.12E-08
Zfp36l1	1.12E-12	0.706072	0.588	0.439	1.28E-08
Arpc4	1.15E-12	-0.35255	0.499	0.645	1.32E-08
Arl6ip1	1.46E-12	-0.34905	0.471	0.627	1.67E-08
Lpcat2	1.52E-12	-0.35987	0.406	0.567	1.74E-08
Pdia6	1.63E-12	-0.35054	0.264	0.439	1.86E-08
Scimp	1.7E-12	-0.3919	0.538	0.687	1.94E-08
Gng5	1.9E-12	-0.40073	0.484	0.647	2.17E-08
Actb	2.15E-12	-0.33491	0.991	0.998	2.45E-08
Spi1	2.87E-12	-0.32752	0.601	0.741	3.28E-08
Mpeg1	3.02E-12	-0.37338	0.881	0.932	3.45E-08
Eif5	3.26E-12	0.35746	0.489	0.305	3.73E-08
Snx18	3.56E-12	0.349558	0.511	0.329	4.06E-08
Tmsb4x	3.67E-12	-0.34955	0.986	0.987	4.19E-08
Rgs2	3.75E-12	0.700905	0.753	0.658	4.29E-08
Jmjd1c	4.46E-12	0.434347	0.561	0.386	5.1E-08
Ybx1	4.55E-12	-0.37568	0.647	0.791	5.2E-08
Sdf2l1	6.13E-12	-0.36613	0.182	0.34	7E-08
Ang	6.9E-12	-0.32756	0.209	0.371	7.88E-08
Lamtor1	7.36E-12	-0.28231	0.155	0.31	8.41E-08
Bsg	8.36E-12	-0.26944	0.121	0.264	9.56E-08
Gdi2	9.22E-12	-0.33513	0.59	0.728	1.05E-07
Scarb2	1.05E-11	-0.3107	0.201	0.361	1.21E-07
Selenop	1.38E-11	-0.34344	0.687	0.804	1.58E-07
Pnp	1.53E-11	-0.34661	0.281	0.444	1.75E-07
Actg1	1.56E-11	0.661034	0.641	0.522	1.78E-07
Arhgdib	1.57E-11	-0.39168	0.495	0.63	1.79E-07
Rgs10	1.59E-11	-0.32825	0.854	0.906	1.82E-07
Arpc1b	1.6E-11	-0.34331	0.578	0.728	1.83E-07
Gm26740	2.29E-11	-0.26314	0.135	0.279	2.62E-07
Irf2bp2	3.47E-11	0.330347	0.406	0.233	3.96E-07
Selplg	3.84E-11	-0.3599	0.293	0.444	4.39E-07
Pik3r1	4.86E-11	0.416199	0.34	0.185	5.56E-07
Swap70	5.17E-11	0.397016	0.397	0.248	5.91E-07
Tra2b	6E-11	0.368186	0.506	0.334	6.86E-07
Cacybp	6.06E-11	0.305438	0.303	0.152	6.92E-07
Ppp1r10	7.19E-11	0.277774	0.373	0.204	8.22E-07

This manuscript has been accepted for publication in Science Immunology. This version has not undergone final editing. Please refer to the complete version of record at www.scienceimmunology.org. The manuscript may not be reproduced or used in any manner that does not fall within the fair use provisions of the Copyright Act without the prior, written permission of AAAS.

Clk1	7.69E-11	0.317453	0.425	0.254	8.79E-07
Eif1	8.39E-11	0.393198	0.813	0.694	9.59E-07
Capza2	1.34E-10	-0.29176	0.66	0.794	1.53E-06
Actr3	1.54E-10	-0.33839	0.836	0.885	1.76E-06
H2-Q7	1.62E-10	0.264289	0.307	0.157	1.85E-06
Arf5	1.77E-10	-0.27255	0.148	0.288	2.02E-06
Adgre1	2.32E-10	-0.32079	0.57	0.692	2.65E-06
Lair1	2.4E-10	-0.33464	0.646	0.77	2.75E-06
Myl6	2.9E-10	-0.34206	0.316	0.475	3.32E-06
Vamp8	2.93E-10	-0.35171	0.215	0.353	3.35E-06
Ctsb	3.96E-10	-0.32218	0.944	0.989	4.52E-06
Bcap31	4.09E-10	-0.26049	0.187	0.331	4.67E-06
Gm2a	5.7E-10	-0.28976	0.446	0.6	6.52E-06
Cd86	5.92E-10	0.323655	0.679	0.543	6.76E-06
Rhob	5.98E-10	0.527487	0.395	0.25	6.83E-06
Ctsa	6.52E-10	-0.34649	0.426	0.548	7.46E-06
AW112010	7.71E-10	-0.48126	0.494	0.624	8.81E-06
Ccnd1	8.05E-10	-0.33939	0.343	0.494	9.2E-06
Ptafr	8.78E-10	0.271195	0.316	0.172	1E-05
Ier3ip1	9.11E-10	-0.25874	0.151	0.284	1.04E-05
Rsrc2	9.45E-10	0.290337	0.426	0.274	1.08E-05
Cflar	9.6E-10	0.346339	0.296	0.162	1.1E-05
Actr2	1E-09	-0.30495	0.512	0.643	1.15E-05
Prdx5	1.16E-09	-0.3727	0.356	0.488	1.33E-05
Lipa	1.38E-09	-0.32003	0.344	0.506	1.57E-05
Asah1	1.47E-09	-0.32955	0.481	0.6	1.68E-05
Atpif1	1.59E-09	-0.36382	0.337	0.478	1.81E-05
Hpgd	1.65E-09	-0.36676	0.598	0.716	1.88E-05
Stap1	1.65E-09	0.359007	0.376	0.229	1.88E-05
Scamp2	2.18E-09	-0.30228	0.284	0.423	2.49E-05
Snrpb	2.28E-09	-0.27491	0.245	0.389	2.6E-05
Brk1	2.31E-09	-0.27053	0.402	0.559	2.64E-05
Tma7	2.38E-09	-0.31377	0.308	0.449	2.72E-05
Atp5f1	2.4E-09	-0.28057	0.181	0.314	2.74E-05
Tpm3	2.76E-09	-0.30612	0.591	0.715	3.16E-05
Ptpn6	4.04E-09	-0.30994	0.403	0.536	4.61E-05
Rrbp1	4.26E-09	-0.33157	0.704	0.796	4.87E-05
Myl12b	4.38E-09	-0.35272	0.423	0.546	5.01E-05
Ppia	5.1E-09	-0.31788	0.824	0.904	5.82E-05
Ppp1ca	5.64E-09	-0.27871	0.215	0.353	6.45E-05
Ypel3	6.31E-09	-0.2504	0.174	0.3	7.21E-05
Arpc2	7.08E-09	-0.2884	0.753	0.823	8.09E-05
Ifnar2	8.01E-09	-0.29244	0.525	0.64	9.15E-05
Ms4a6c	9.23E-09	-0.32311	0.653	0.75	0.000105

This manuscript has been accepted for publication in Science Immunology. This version has not undergone final editing. Please refer to the complete version of record at www.scienceimmunology.org. The manuscript may not be reproduced or used in any manner that does not fall within the fair use provisions of the Copyright Act without the prior, written permission of AAAS.

Ptpn18	1.11E-08	-0.29207	0.594	0.707	0.000127
Cyth4	1.2E-08	-0.3258	0.584	0.684	0.000137
Atp6v1g1	1.44E-08	-0.26796	0.268	0.407	0.000164
Csf2ra	1.74E-08	-0.3017	0.687	0.752	0.000199
Gsn	1.79E-08	-0.27594	0.257	0.389	0.000204
Rab7b	1.97E-08	0.268746	0.327	0.196	0.000226
Calm1	2.03E-08	-0.30119	0.722	0.836	0.000232
Grcc10	2.04E-08	-0.26694	0.227	0.358	0.000233
Tapbp	2.04E-08	-0.26191	0.245	0.381	0.000234
Pomp	2.16E-08	-0.26343	0.334	0.473	0.000247
Cebpa	2.42E-08	0.385191	0.399	0.271	0.000277
Arpc5	2.55E-08	-0.30949	0.402	0.528	0.000291
Cyp4f18	3.16E-08	-0.30117	0.3	0.421	0.000361
Hcls1	3.91E-08	-0.28124	0.446	0.561	0.000447
Arpc3	4.52E-08	-0.28757	0.714	0.802	0.000516
Tmem14c	4.66E-08	-0.28882	0.204	0.324	0.000533
Tnfsf13b	4.68E-08	-0.28188	0.317	0.449	0.000535
Tsc22d3	5E-08	0.319254	0.257	0.139	0.000572
Stk17b	5.15E-08	0.2577	0.376	0.238	0.000589
Fyb	5.34E-08	-0.27047	0.877	0.917	0.00061
Syngr2	5.87E-08	-0.25318	0.268	0.404	0.000671
Camk1	6.03E-08	-0.31191	0.349	0.468	0.00069
Rhoa	6.36E-08	-0.28133	0.631	0.736	0.000726
Cd180	6.82E-08	-0.27445	0.288	0.418	0.00078
Gatm	7.24E-08	-0.32057	0.604	0.71	0.000827
Fam49b	7.59E-08	-0.25044	0.601	0.7	0.000867
Sf3b1	7.94E-08	0.284175	0.624	0.485	0.000907
Hivep2	8.12E-08	0.309287	0.32	0.196	0.000928
Il10rb	8.52E-08	-0.29947	0.353	0.473	0.000974
Rbx1	9.7E-08	-0.30274	0.275	0.4	0.001108
Plk3	9.82E-08	0.265756	0.301	0.178	0.001122
Abhd12	9.89E-08	-0.27266	0.359	0.481	0.00113
Cnih4	9.92E-08	-0.27788	0.191	0.305	0.001133
Tmed10	1.02E-07	-0.27648	0.284	0.415	0.001161
Slc11a1	1.11E-07	-0.27086	0.277	0.404	0.001269
Lrrc25	1.21E-07	-0.25903	0.27	0.394	0.001385
Twf2	1.31E-07	-0.2558	0.247	0.37	0.001499
Rnf130	1.33E-07	-0.25264	0.288	0.412	0.001514
Lilra5	1.51E-07	-0.29573	0.605	0.689	0.001725
Marcks	1.64E-07	-0.26428	0.825	0.896	0.001868
H2afz	1.86E-07	-0.26756	0.518	0.647	0.002122
Taldo1	2.03E-07	-0.27898	0.184	0.297	0.002322
Sat1	2.07E-07	0.642558	0.845	0.809	0.002369
Ftl1	2.2E-07	0.297543	0.98	0.994	0.002513

This manuscript has been accepted for publication in Science Immunology. This version has not undergone final editing. Please refer to the complete version of record at www.scienceimmunology.org. The manuscript may not be reproduced or used in any manner that does not fall within the fair use provisions of the Copyright Act without the prior, written permission of AAAS.

Slfn2	2.5E-07	0.490123	0.478	0.363	0.002852
Rac1	3.13E-07	-0.2675	0.409	0.527	0.003575
Ighm	3.24E-07	-0.26554	0.436	0.556	0.003704
Lamp2	3.29E-07	-0.26645	0.598	0.697	0.003763
Slco2b1	3.34E-07	-0.27254	0.303	0.415	0.003815
Eid1	4.09E-07	-0.29271	0.429	0.545	0.004678
Oaz1	4.45E-07	-0.31837	0.436	0.553	0.005081
Prpf4b	4.51E-07	-0.28418	0.207	0.319	0.005156
Rbpj	4.7E-07	0.251843	0.545	0.417	0.005373
Qpct	4.83E-07	-0.30373	0.451	0.559	0.005525
Psma7	4.85E-07	-0.25154	0.184	0.295	0.005537
Lgals3bp	5.1E-07	-0.27237	0.324	0.436	0.005833
Cfl1	5.19E-07	-0.25275	0.788	0.864	0.005936
Slc15a3	5.25E-07	0.299727	0.64	0.512	0.006002
Rbm39	6.48E-07	0.2598	0.735	0.626	0.007408
Gpr183	6.71E-07	0.514461	0.549	0.457	0.007668
Bmyc	6.89E-07	-0.28717	0.359	0.462	0.00787
2410006H16Rik	9.18E-07	0.274773	0.605	0.465	0.01049
Fcgr4	1.04E-06	-0.28657	0.344	0.452	0.011883
Ifi207	1.04E-06	0.318166	0.646	0.54	0.01189
Rnaset2a	1.08E-06	-0.28072	0.38	0.483	0.01233
Ywhab	1.11E-06	-0.26086	0.288	0.394	0.012656
Pf4	1.11E-06	-0.26526	0.202	0.314	0.012734
Slc29a3	1.67E-06	-0.28062	0.455	0.535	0.01913
Sem1	2.61E-06	-0.27581	0.445	0.543	0.02982
Ccr1	2.78E-06	-0.28382	0.214	0.313	0.031767
Atp6v1f	3.2E-06	-0.26074	0.317	0.418	0.036523
Dleu2	3.22E-06	0.264731	0.737	0.639	0.036781
Gns	3.26E-06	-0.26327	0.36	0.457	0.037305
Axl	3.29E-06	-0.30756	0.518	0.585	0.037621
Fuca1	4.91E-06	-0.25835	0.3	0.402	0.05614
Pitpna	5.13E-06	-0.25253	0.32	0.423	0.0586
Hmgb2	6.16E-06	0.43544	0.631	0.546	0.070415
Maf	6.24E-06	-0.2884	0.291	0.392	0.071351
Cd72	7E-06	-0.25121	0.699	0.778	0.079973
Ifit2	1.46E-05	0.644048	0.254	0.165	0.167113
Tnfaip8	2.12E-05	0.266167	0.585	0.489	0.242697
Atp6v0c	2.21E-05	0.285019	0.749	0.692	0.253001
Ntpcr	2.59E-05	-0.25762	0.486	0.562	0.296309
Mbnl1	3.91E-05	-0.25267	0.624	0.694	0.446355
Canx	5.23E-05	-0.25187	0.291	0.382	0.597214
Zfp36l2	7.54E-05	0.356254	0.571	0.483	0.862045
Isg15	9.01E-05	0.703088	0.268	0.18	1
Cited2	9.73E-05	0.571634	0.373	0.298	1

[#] This manuscript has been accepted for publication in Science Immunology. This version has not undergone final editing. Please refer to the complete version of record at www.scienceimmunology.org. The manuscript may not be reproduced or used in any manner that does not fall within the fair use provisions of the Copyright Act without the prior, written permission of AAAS.

Lgals3	0.000226	-0.28013	0.222	0.313	1
Lpl	0.00049	-0.30495	0.4	0.47	1
Cox4i1	0.000737	-0.25619	0.268	0.344	1
Ifi27l2a	0.000857	-0.41189	0.268	0.34	1
Neurl3	0.001406	0.276889	0.458	0.391	1
Sap30	0.002254	0.268143	0.35	0.284	1
Ccl12	0.002635	0.558826	0.356	0.292	1
Hspa5	0.002665	0.250805	0.72	0.632	1
Acsl1	0.00395	0.402176	0.436	0.384	1

Table S2. List of differentially expressed genes (DEG) in subsets A-D. The number of DEGs comparing naïve and day 3 post irradiation in annotated macrophages from scRNA-seq was identified (expressed in >25% of cells/cluster, logFC >0.25 and adj p <0.05) with the top 10 genes ranked by logFC.

Cluster	DEG	Gene	LogFC	Adj p value
A	219	*Jund*	1.02	1E-34
		H2-Eb1	0.82	4E-75
		Cd74	0.80	2E-63
		H2-Aa	0.76	1E-60
		Dleu2	0.75	2E-49
		H2-Ab1	0.72	2E-51
		Txnip	0.64	1E-57
		Hnrnpa2b1	0.59	7E-36
		Laptm4a	0.56	1E-64
		Hspa1b	0.54	2E-04
B	232	*H2-Aa*	0.89	7E-44
		H2-Eb1	0.89	1E-46
		Dleu2	0.86	9E-29
		Cd74	0.85	2E-34
		H2-Ab1	0.73	2E-32
		Malat1	0.63	8E-16
		Cx3cr1	0.63	2E-19
		AY036118	0.62	2E-13
		Mef2c	0.62	7E-19
		Tgfbr1	0.56	1E-17
C	15	*H2-Ab1*	2.26	3E-02
		H2-Aa	2.08	2E-02
		Btg2	1.99	1E-02

This manuscript has been accepted for publication in Science Immunology. This version has not undergone final editing. Please refer to the complete version of record at www.scienceimmunology.org. The manuscript may not be reproduced or used in any manner that does not fall within the fair use provisions of the Copyright Act without the prior, written permission of AAAS.

		Rrbp1	1.28	1E-03
		Hnrnpa2b1	1.15	5E-02
		Mycbp2	0.73	7E-03
		Ftl1	-0.75	4E-02
		C1qb	-0.84	6E-04
		Fcer1g	-0.87	1E-03
		C1qa	-0.89	2E-02
**D**	1	*Egln2*	-0.63	1E-03

Table S3. Mouse strains used in this study, their source and relevant identifiers.

**Strain**	**Source**	**Identifier**
C57BL/6J	Charles River	
C57BL/6J CD45.1	University of Edinburgh	
C57BL/6J CD45.2^{+}	University of Edinburgh	
C57BL/6J CD45.1/2^{+}	University of Edinburgh	
Cdh5$^{Cre\text{-}ERT2}$.Rosa26$^{LSL\text{-}CAG\text{-}tdT}$.Cx3cr1$^{gfp/+}$	University of Edinburgh	*(79)*
Csf1r^{FRed}	C. Pridans, University of Edinburgh	(*21*)
Csf1r$^{\Delta FIRE/\Delta FIRE}$	C. Pridans, University of Edinburgh	(22)
Csf2ra$^{-/-}$	C. Schneider, University of Zurich	(80)
Cx3cr1$^{gfp/+}$	Originally obtained from Prof. J. Pollard, University of Edinburgh	*(81)*
Cx3cr1 $^{tm2.1(cre/ERT2)Jung}$	Jackson Laboratories (JAX)	Stock ID: 020940
Il34$^{LacZ/LacZ}$	B. Becher, University of Zurich	(*24*)
Mafb^{Cre}.Cx3cr1$^{LSL\text{-}DTR}$	N. Mabbott, University of Edinburgh, originally obtained from Jackson Laboratories (JAX)	***(82)* (Mafb-Cre)** ***(83)* (*Cx3cr1$^{LSL\text{-}DTR}$*)** **Stock IDs: 025629 and 029664**
Ms4a3$^{Cre/+}$.Rosa26$^{LSL\text{-}CAG\text{-}tdTomato/+}$	F. Ginhoux, ASTAR	(*35*)
Rosa26$^{LSL\text{-}tdRFP}$ (Gt(Rosa)26Sor ^{tm1Hjf})	E. Dzierzak, University of Edinburgh	(84)

This manuscript has been accepted for publication in Science Immunology. This version has not undergone final editing. Please refer to the complete version of record at www.scienceimmunology.org. The manuscript may not be reproduced or used in any manner that does not fall within the fair use provisions of the Copyright Act without the prior, written permission of AAAS.

Table S4. Antibodies used for flow cytometry.

Antibody	Clone	Supplier	Cat #	Dilution	RRID
Mouse BrdU PE	Bu20a	Biolegend	339812	1:200	AB_1626186
Rat CD11b APC/Fire 750	M1/70	Biolegend	101262	1:200	AB_2572122
Rat CD11b PE	M1/70	Biolegend	101207	1:200	AB_312790
Rat CD11b APC-Fire750	M1/70	Biolegend	101262	1:200	AB_2572122
Armenian Hamster CD11c BV785	N418	Biolegend	117336	1:200	AB_2565268
Rat CD115 APC	AFS98	Biolegend	135510	1:200	AB_2085221
Rat CD14 Superbright 600	Sa2-8	Invitrogen	63-0141-82	1:200	AB_2762769
CD14 BV785	Sa14-2	Biolegend	123337	1:200	AB_2888880
Rat CD16/CD32 TruStain FcX	S17011E	Biolegend	156603	1:1000	AB_2783137
Rat CD45 APC-Cy7	30-F11	BD Biosciences	557659	1:200	AB_396774
Rat CD45 BV510	30-F11	Biolegend	103138	1:200	AB_2563061
Mouse CD45.1 AF488	A20	Biolegend	110718	1:200	AB_492862
Mouse CD45.1 BV510	A20	Biolegend	110741	1:200	AB_2563378
Mouse CD45.2 AF700	104	Biolegend	109822	1:200	AB_493731
CD63 PE	NVG-2	Biolegend	143903	1:200	AB_11203532
Mouse CD64 BV421	X54-5/7.1	Biolegend	139309	1:200	AB_2562694
Rat CD86 FITC	GL1	Invitrogen	11-0862-85	1:200	AB_465149
Rat CD163 PE	S15049F	Biolegend	156704	1:200	AB_2860724
Mouse CD206 PE/Cy7	MR6F3	Invitrogen	25-2061-82	1:200	AB_2637424
Rat CD206 eFluor 450	MR6F3	Invitrogen	48-2061-80	1:200	AB_2762720

This manuscript has been accepted for publication in Science Immunology. This version has not undergone final editing. Please refer to the complete version of record at www.scienceimmunology.org. The manuscript may not be reproduced or used in any manner that does not fall within the fair use provisions of the Copyright Act without the prior, written permission of AAAS.

Rat CD209 PE	LWC06	Invitrogen	12-2092-82	1:200	AB_657702
Mouse CX3CR1 PE	SA011F11	Biolegend	149005	1:200	AB_2564314
Mouse CX3CR1 Biotin	SA011F11	Biolegend	149018	1:200	AB_2565701
Mouse CX3CR1 APC	SA011F11	Biolegend	149007	1:200	AB_2564491
Rat F4/80 PE	BM8	Biolegend	123110	1:200	AB_893486
Rat F4/80 PE/Cy5	BM8	Biolegend	123111	1:200	AB_893494
Rat F4/80 APC	BM8	Invitrogen	17-4801-82	1:200	AB_2784648
FRβ APC	10/FR2	Biolegend	153306	1:200	AB_2721313
Ki67 FITC	REA183	Miltenyi	130-117-803	1:200	AB_2733584
Mouse Ly6C APC eFluor 780	HK1.4	Invitrogen	47-5932-82	1:200	AB_2573992
Ly6C AF450	HK1.4	eBioscience	48-5932-82	1:200	AB_10805519
Rat Ly6C PerCP/Cy5.5	HK1.4	Biolegend	128012	1:200	AB_1659241
Rat Ly6G Biotin	1A8	Biolegend	127604	1:200	AB_1186108
Rat Ly6G FITC	1A8	Biolegend	127606	1:200	AB_1236494
Rat MerTK PE	2B10C42	Biolegend	151506	1:200	AB_2617037
Rat MHC II AF700	M5/114.15.2	Biolegend	107622	1:200	AB_493727
Rat MHC II PE/Cy5	M5/114.15.2	Invitrogen	15-5321-82	1:200	AB_468800
Mouse SiglecF FITC	REA798	Miltenyi	130-112-178	1:200	n/a
Streptavidin BV650	n/a	Biolegend	405231	1:10000	n/a
Rat Tim4 PE/Cy7	RMT4-54	Biolegend	130010	1:200	AB_2565719

Table S5. Primary antibodies used for immunofluorescent staining.

Antibody	Clone	Species	Supplier	Cat #	Dilution	RRID
53BP1		Rabbit	Novus	NB100-304	1:1200	AB_1659863
CASP3	5A1E	Rabbit	Cell Signaling	9664L	1:300	AB_2070042
Claudin 10		Rabbit	Life Technologies	38-8400	1:100	AB_2533386
AQP5[ω]		Rabbit	Millipore	AB3559	1:200	AB_2141915
AQP5	EPR3747	Rabbit	Abcam	ab92320	1:200	AB_2049171
CD31		Goat	BioTechne	AF3628	1:300	AB_2161028
CD31-EF450	390	Rat	Thermo Fisher	48-0311-82	1:200	AB_10598807
CD163	TNKUPJ	Rat	Thermo Fisher	14-1631-82	1:800	AB_2716934
CD206-APC	C068C2	Mouse	Biolegend	141708	1:200	AB_10896057
CD45		Goat	R&D Systems	AF114	1:200	AB_442146
E-Cadherin	ECCD2	Rat	Life Technologies	13-1900	1:300	AB_2533005
EpCAM-AF594	G8.8	Rat	Biolegend	118222	1:400	AB_2563322
F4/80	A3-1	Rat	Abcam	ab6640	1:200	AB_1140040
GFP		Goat	Abcam	ab6662	1:600	AB_305635
IBA1		Rabbit	Antibodies Online	ABIN2857032	1:200	
Ki67	SolA15	Rat	Invitrogen	14-5698-82	1:200	AB_10854564
MIST1	D7N4B	Rabbit	Cell Signaling	14896T	1:100	AB_2798639
TUJ1		Rabbit	Abcam	ab18207	1:400	AB_444319

[#] This manuscript has been accepted for publication in Science Immunology. This version has not undergone final editing. Please refer to the complete version of record at www.scienceimmunology.org. The manuscript may not be reproduced or used in any manner that does not fall within the fair use provisions of the Copyright Act without the prior, written permission of AAAS.

ω This antibody has been discontinued

Table S6. Primer sequences used for qPCR.

Gene	Forward primer	Reverse primer
Gapdh	AGGTCGGTGTGAACGGATTTG	TGTAGACCATGTAGTTGAGGTCA
Amy1	ATCACAGTGCTGACAGAATCCATATTTG	TTTCATTTGGTTTCAATTTCTCTTTCGTTC
Aqp5	TCTACTTCTACTTGCTTTTCCCCTCCTC	CGATGGTCTTCTTCCGCTCCTCTC
Bax	TGAAGACAGGGGCCTTTTTG	AATTCGCCGGAGACACTCG
Ccl8	TCTACGCAGTGCTTCTTTGCC	AAGGGGGATCTTCAGCTTTAGTA
Cdh1	GACTGGAGTGCCACCACCAAAGAC	CGCCTGTGTACCCTCACCATCGG
Csf1	ATGAGCAGGAGTATTGCCAAGG	TCCATTCCCAATCATGTGGCTA
Csf1r	TGTCATCGAGCCTAGTGGC	CGGGAGATTCAGGGTCCAAG
Cxcl2	CCAACCACCAGGCTACAGG	GCGTCACACTCAAGCTCTG
Ifitm3	CCCCCAAACTACGAAAGAATCA	ACCATCTTCCGATCCCTAGAC
Il1a	CGAAGACTACAGTTCTGCCATT	GACGTTTCAGAGGTTCTCAGAG
Il6	CCGGAGAGGAGACTTCACAG	TCCACGATTTCCCAGAGAAC
mKi67	CATACCTGAGCCCATCACCA	GCTTTGCTGCATTCCGAGTA
Nlrp3	ATTACCCGCCCGAGAAAGG	TCGCAGCAAAGATCCACACAG
Prol1	CACCTAAGCCTAGCACCTCTA	ACTTCCAAAACACTTCCGCAAAT
Sox10	ATCAGCCACGAGGTAATGTCCAAC	ACTGCCCAGCCCGTAGCC

This manuscript has been accepted for publication in Science Immunology. This version has not undergone final editing. Please refer to the complete version of record at www.scienceimmunology.org. The manuscript may not be reproduced or used in any manner that does not fall within the fair use provisions of the Copyright Act without the prior, written permission of AAAS.

Tnfa	CATCTTCTCAAAATTCGAGTGACAA	TGGGAGTAGACAAGGTACAACCC

Table S7. Antibodies used for fluorescence activated cell sorting (FACS).

Antibody	Clone	Supplier	Cat #	Dilution	RRID
Rat CD11b APC/Fire 750	M1/70	Biolegend	101262	1:200	AB_2572122
Rat CD163 PE	S15049F	Biolegend	156704	1:200	AB_2860724
Rat CD19 FITC	6D5	Biolegend	115505	1:200	
Rat CD206 eFluor 450	MR6F3	Invitrogen	48-2061-80	1:200	AB_2762720
Rat CD3 FITC	17A2	Biolegend	100204	1:200	
Rat CD31 PE-Cy7	MEC13.3	Biolegend	102523	1:800	AB_2572181
Rat CD326 PE	G8.8	Biolegend	118205	1:4000	AB_1134176
Rat CD45 BV510	30-F11	Biolegend	103138	1:200	AB_2563061
Rat F4/80 APC	BM8	Invitrogen	17-4801-82	1:200	AB_2784648
Rat Ly6G FITC	1A8	Biolegend	127606	1:200	AB_1236494
Mouse SiglecF FITC	REA798	Miltenyi	130-112-178	1:200	n/a

This manuscript has been accepted for publication in Science Immunology. This version has not undergone final editing. Please refer to the complete version of record at www.scienceimmunology.org. The manuscript may not be reproduced or used in any manner that does not fall within the fair use provisions of the Copyright Act without the prior, written permission of AAAS.
